# A robust biostatistical method leverages informative but uncertainly determined qPCR data for biomarker detection, early diagnosis, and treatment

**DOI:** 10.1371/journal.pone.0263070

**Published:** 2022-01-31

**Authors:** Wei Zhuang, Luísa Camacho, Camila S. Silva, Michael Thomson, Kevin Snyder

**Affiliations:** 1 Division of Bioinformatics and Biostatistics, National Center for Toxicological Research, U.S. Food and Drug Administration, Jefferson, Arkansas, United States of America; 2 Division of Biochemical Toxicology, National Center for Toxicological Research, U.S. Food and Drug Administration, Jefferson, Arkansas, United States of America; 3 Office of New Drugs, Center for Drug Evaluation and Research, U.S. Food and Drug Administration, Silver Spring, Maryland, United States of America; Universidade Lisboa, Instituto superior Técnico, PORTUGAL

## Abstract

As a common medium-throughput technique, qPCR (quantitative real-time polymerase chain reaction) is widely used to measure levels of nucleic acids. In addition to accurate and complete data, experimenters have unavoidably observed some incomplete and uncertainly determined qPCR data because of intrinsically low overall amounts of biological materials, such as nucleic acids present in biofluids. When there are samples with uncertainly determined qPCR data, some investigators apply the statistical complete-case method by excluding the subset of samples with uncertainly determined data from analysis (CO), while others simply choose not to analyze (CNA) these datasets altogether. To include as many observations as possible in analysis for interesting differential changes between groups, some investigators set incomplete observations equal to the maximum quality qPCR cycle (MC), such as 32 and 40. Although straightforward, these methods may decrease the sample size, skew the data distribution, and compromise statistical power and research reproducibility across replicate qPCR studies. To overcome the shortcomings of the existing, commonly-used qPCR data analysis methods and to join the efforts in advancing statistical analysis in rigorous preclinical research, we propose a robust nonparametric statistical cycle-to-threshold method (CTOT) to analyze incomplete qPCR data for two-group comparisons. CTOT incorporates important characteristics of qPCR data and time-to-event statistical methodology, resulting in a novel analytical method for qPCR data that is built around good quality data from all subjects, certainly determined or not. Considering the benchmark full data (BFD), we compared the abilities of CTOT, CO, MC, and CNA statistical methods to detect interesting differential changes between groups with informative but uncertainly determined qPCR data. Our simulations and applications show that CTOT improves the power of detecting and confirming differential changes in many situations over the three commonly used methods without excess type I errors. The robust nonparametric statistical method of CTOT helps leverage qPCR technology and increase the power to detect differential changes that may assist decision making with respect to biomarker detection and early diagnosis, with the goal of improving the management of patient healthcare.

## Introduction

Recent studies have indicated the value of informative but uncertainly determined qPCR data in patient and disease management while using human biofluids, such as serum, to assess levels of pathogens, nucleic acids, and tumor cells [1, 2]. Research on the levels of biological materials in biofluids holds promise to identify biomarkers for early detection of diseases and to optimize treatment regimens, such as treatment dosage, frequency, and duration [1, 3–5]. For the context of this study, investigators have faced uncertainly determined qPCR observations due to the overall low levels of nucleic acids, such as in biofluids, in addition to certainly quantified qPCR data [1, 2, 3, 6]. While levels of molecular targets may be uncertainly determined due to experimental errors, in this study we focus on scenarios with errorless pre-PCR preparation and reliable real-time qPCR reactions, as these scenarios represent good and proper practice of qPCR technologies [7]. Even under the carefully optimized and properly conducted studies, targets with absent or low levels in biological samples (e.g., serum) are inherently difficult to measure and certainly determine. However, interesting differential changes may involve absent or low molecular levels, for example, the transmission from large or moderate levels to absent or low levels. Technically, the true quantification cycle Cq values of these measurands with low levels are censored at a known censoring point C_1_, e.g., 32 or 40, and they are often reported as undetermined by commercial qPCR products [8–12]. The phenomenon is similar to administrator censoring at the end of a survival or time-to-event study, as it occurs at a known ending point C_1_, the end of a qPCR experiment [13, 14]. Other types of censoring, e.g., participants dropout without experiencing an event of interest before the end of the study, are not relevant to qPCR experiments with errorless pre-PCR preparation and reliable real-time qPCR reactions [13, 14]. For simplicity, we use “uncertain qPCR data” to refer to those qPCR observations that are uncertainly determined in the scenarios with errorless pre-PCR preparation and reliable real-time qPCR reactions. In this study, we designed and evaluated a nonparametric statistical cycle-to-threshold method (CTOT) to improve power in detecting interesting differential changes with uncertain but informative qPCR data between groups of interest. While maintaining the type I error rate, the robust statistical method with improved power may help leverage qPCR technology, enhance screening and detection of biomarker candidates, and contribute to treatment optimization and precision medicine.

As an application of the widely-used PCR technique, qPCR has been used to measure gene expression, identify biomarkers, and understand biological mechanisms, e.g., toxicity, cancer, and cardiovascular disease, among many others [1–3, 15–17]. qPCR technology is used to assess the levels of a molecular target via the continuous monitoring and quantification of a fluorescence signal that is proportional to the input of the DNA of interest in the PCR exponential phase. Among other applications, qPCR technology has been widely used to measure the levels of RNA transcripts, e.g., virus RNAs, messenger RNAs (mRNAs) and microRNAs. qPCR is a popular method for development of diagnostic assays due to its high performance [18–20]. The number of publications on the topic of qPCR and microRNAs has increased from fewer than 1,500 in 2016 to more than 2,500 in 2020 in the Web of Science, as illustrated in S1 Fig [21]. Technically, the RT-qPCR method uses complementary DNAs (cDNAs) reverse-transcribed from RNA for subsequent qPCR amplification, while the qPCR method directly uses DNA for qPCR amplification. In this paper, the term “qPCR data” refers to data resulting from either qPCR or RT-qPCR reactions.

Like many bioanalytical technologies, a qPCR assay is usually established with a limit of detection (LOD) and a lower limit of quantification (LLOQ) to detect or quantify the initial concentration of molecular input [1, 22, 23]. The terms of LOD and LLOQ are conceptualized as the minimum concentration of DNA that can be accurately detected and the minimum concentration that can be accurately quantified, respectively [24, 25]. LLOQ is usually larger than LOD [1, 24, 25]. For example, the LOD of plasma hepatitis C virus RNA in the Roche COBAS TaqMan HCV 2.0 assay was considered 9.3–10 international units per mL (IU/mL), while the LLOQ was larger and considered 25 IU/mL [1]. In addition, a qPCR assay may have a limit of blank (LOB), which is the highest concentration expected to be observed when replicates of a blank sample containing no targets of interest are measured [24, 25]. LOB is usually set to a high percentile of the distribution of observed concentrations of blank samples, e.g., 95^th^ percentile [24, 25]. LOD is then set so that only a small proportion of its distribution is below LOB. For example, LOD is often set at 5^th^ percentile of the distribution of its observed concentrations [24, 25]. Because of intrinsic measurement limitations of LLOQ, LOD, and LOB, rare or completely absent nucleic acids tend not to be certainly quantified with exact values in qPCR [8–12]. Biologically, the effect of the treatment may decrease the concentration of a molecular target, e.g. virus RNA, to null or to a level below LLOQ or LOD [1]. Conversely, the steady levels of a molecular target under untreated conditions may be below LLOQ or LOD. Technically, the input amount of DNA template for a qPCR assay may be limited by the availability of the biological sample (e.g., serum) itself and because of potential issues with inhibition of the qPCR from the carryover of components, such as salts, in the DNA template solution [22]. These situations result in incomplete or uncertain qPCR quantification with biological, clinical, and/or technical relevance.

**Fig 1:**
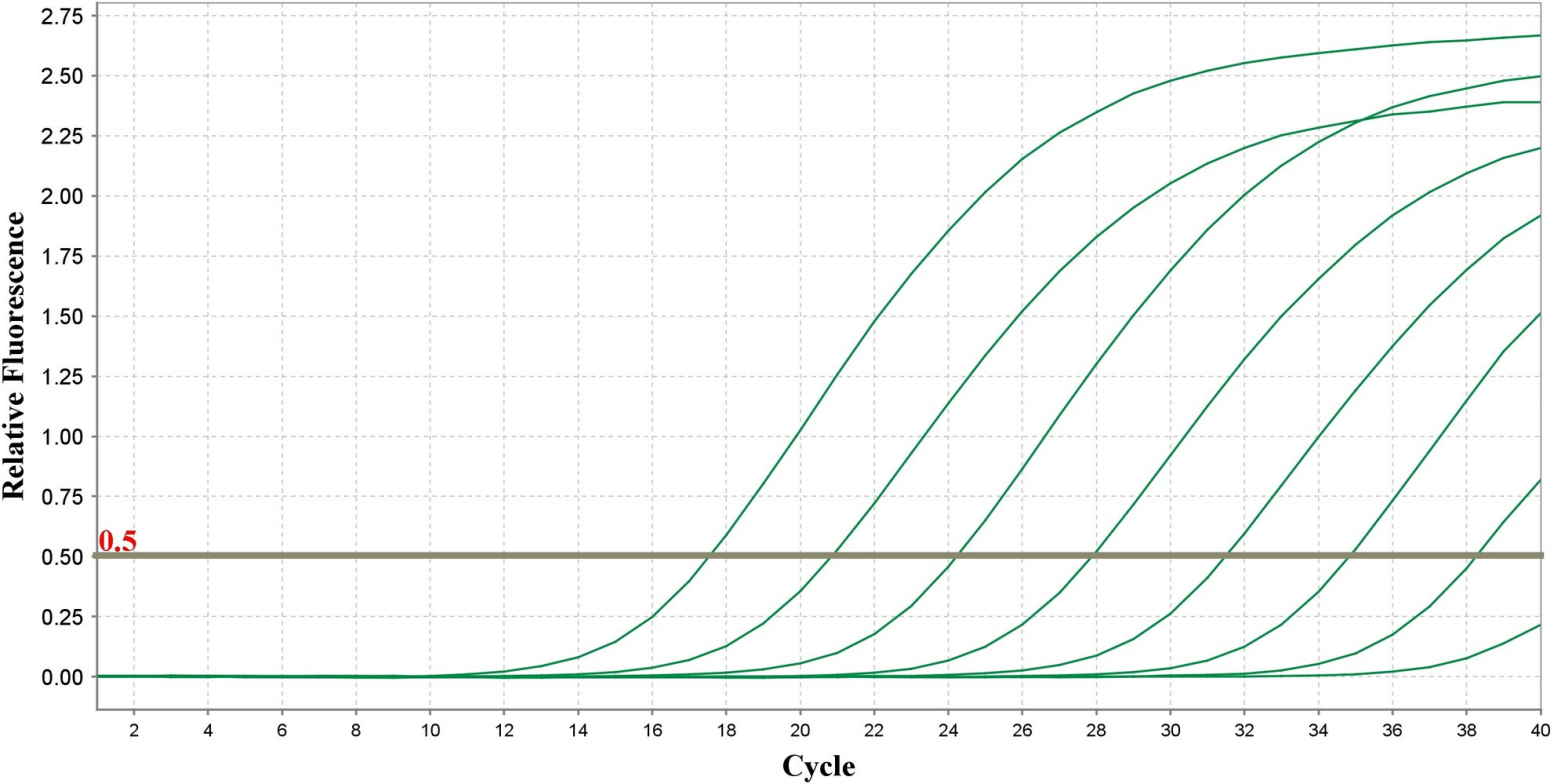
Amplification curves of qPCR reactions. In the example, five molecular targets reached the threshold of 0.5 before the 32^nd^ cycle, i.e., Cq < 32, while two reached the threshold between the 32^nd^ and the 40^th^ cycle. The eighth molecular target did not reach the threshold by the 40^th^ cycle, i.e., Cq > 40. QuantStudio Real-Time PCR software version 1.3 (Applied Biosystems by Thermo Fisher Scientific) was used to create the figure.

As illustrated in Fig 1, the initial input of a molecular target is continuously amplified through cycles until the pre-set maximum cycle, e.g., 40, is reached. In literature, the notations of Cq and Ct are often used to denote the amplification cycle number that intersects the fluorescence threshold [20, 26–28]. For simplicity, we use the notation of Cq instead of Ct to follow the MIQE (Minimum Information for Publication of Quantitative Real-Time PCR Experiments) guidelines in this study [20]. In general, the lower the initial concentrations, the higher the Cq values as more amplification cycles are needed to reach the fluorescence threshold (Fig 1). Because of LLOQ, LOD, and LOB, many researchers specify contextual Cq cut-offs (denoted as C_1_) varying between 32 and 50 [2, 22, 29, 30]. Specifically, because of the inverse relationship between initial concentrations and the Cq values, the original Cq values less than C_1_ are considered certain for contextual purposes. For others, the quantification is considered uncertain with true values hidden in a range (e.g., *C*_*q*_ ≥ *C*_1_) or plausibly affected with non-negligible factors (e.g., incorrect dilutions of DNA template and DNA-oligonucleotide binding issues).

Many differential changes in qPCR data have been detected with inferential statistical methods, which make inferences about the study/target populations using data collected from samples through study designs. The inferences and generalizations are valid if the underlying statistical assumptions are not violated. Informative but uncertain qPCR observations may challenge the underlying assumptions of commonly used statistical methods to analyze qPCR data, e.g. t-tests and its variant methods of applying the complete-observation method (CO) and setting incomplete observations equivalent to the maximum quality number of qPCR cycles (MC) [31]. Besides CO and MC, the method of choosing not to analyze (CNA) targets with incomplete observations has been used in the context of two-group comparisons with qPCR data [20, 31]. In the current study, we designed a cycle-to-threshold method (CTOT) that was enriched by the time-to-event statistical framework and important characteristics of qPCR data. These characteristics include the principle underlying qPCR data normalization, the utilization of a fluorescence threshold in a qPCR experiment, and the potential for a few or some observations to be censored at the end of a well-conducted qPCR experiment [32, 33]. It is worth noting that, in this study, qPCR data normalization does not refer to the alignment of observed data to a statistical distribution, such as a normal distribution [34, 35]. It refers to the process to correct unwanted variance in Cq values before statistical analysis, which has been implemented in the widely used 2(-Delta Delta C(T)) method for real-time quantitative PCR analysis [27, 31]. The principle of such normalization has been widely utilized in many biomedical research areas, such as sequencing data analysis and neuroimage analysis [36, 37]. We used simulations to investigate the performance of CTOT, MC, CO, and CNA methods and explored applicational aspects of CTOT with respect to biomarker detection in liquid biopsies when low or absent levels of molecules, e.g., microRNAs, indicate biological processes of interest, e.g., pathogenic processes, normal biological processes, and biological responses to an exposure or therapeutic treatment. The performance of the CTOT, MC, and CO methods were explored in terms of statistical reproducibility or potential research reproducibility as statistical reproducibility is often related to research reproducibility [33, 38]. In simulations, we can simulate many replicates of samples in a target population. The abundant replicates are used to evaluate the abilities of a statistical method to draw inferences with different replicates of the same underlying truth. The maintenance of a low nominal type I error rate, such as 0.05 and 0.005, and achievement of a high statistical power, such as 80% and 90%, are two manifestations of potential research reproducibility of a statistical method [38, 39]. Specifically, a type I error is the probability that researchers reject a true null hypothesis. An example of a type I error is that researchers determine a difference when there is no difference between treated and untreated subjects. Correspondingly, statistical power is the probability that researchers reject a false null hypothesis. In this study, we obtained the empirical type I error rate and statistical power of a statistical method in simulations. Finally, we applied CTOT to perform sensitivity analysis and statistically verify biomarker discoveries in a previously published dataset of circulating microRNAs with potentially uncertain Cq observations [3].

Our simulations and applications showed that CTOT could be more robust and more powerful in many situations, compared with existing methods, such as the CNA, CO, and MC methods. It could improve the power of identifying informative differential changes between control and treated groups over the existing methods without excess type I errors, when minute sizes and low levels of molecular targets are involved in the differential changes. In the application, CTOT detected potentially differential expression that could be overlooked otherwise by existing methods (CNA, MC, and CO). Therefore, we propose CTOT, the robust nonparametric cycle-to-threshold method, to leverage or compensate uncertain but informative qPCR data and leverage their potential for biomarker detection, early diagnosis, or treatment optimization.

## Materials and methods

### CTOT

In this study, we designed the CTOT method to analyze qPCR data with certain and uncertain quantification, which were collected for group comparison to test the null hypothesis of equality, such as equal gene expression across two groups. The certain and uncertain qPCR data can be organized and tabulated as illustrated in Table 1. In Table 1, *Y*_*(ijk)*_ denotes the Cq value reported by a qPCR assay for molecular target *j* (*j* = 1, 2, …, or *g*) of sample *i* (*i* = 1, 2, …, or *n*) in group *k* (*k* = 1 or 2). The common outcome notation of Y in the statistics field is used to denote Cq for notation simplicity and generalizability, since Cq is one of the two outcomes in the study setting. Likewise, a common notation of an indicator variable in the statistics field is used to denote whether Cq < C_1_. *φ*_*(ijk)*_ denotes I(*Y*_*(ijk)*_ < C_1_), which is a binary variable that takes the value of 1 when the Cq is smaller than C_1_ and takes the value of 0 otherwise. *φ*_*(ijk)*_ is another outcome in the study setting and is used to incorporate the information on whether the true Cq is observed with sufficient certainty. For datasets with just certain observations, *φ*_*(ijk)*_ is 1 for any molecular target *j* of sample *i* with treatment *k* and can be ignored in analysis to test the null hypothesis of no relationship between the treatment (or exposure) and the levels of a molecular target. In addition, normalization is used to correct unwanted variance in *Y*_*(ijk)*_, due to unwanted factors, such as different molecular target input amounts, which may cause samples in the same group to reach the fluorescence threshold at different cycles [27, 40]. Δ*Y*_*(ijk)*_ denotes the normalized Cq with unwanted variance (e.g., the input variance) corrected via the equation of Δ*Y*_*(ijk) =*_ *Y*_*(ijk)*_*–Y*_*(irk)*_, where *Y*_*(irk)*_ denotes the well-observed Cq value of the normalizer of sample *i* (*i* = 1, 2, …, or *n*) in group *k* (*k* = 1 or 2).

**Table 1:** Symbolic qPCR data set.

Group	Outcome	Normalized Outcome
1	(*Y*_*(1j1)*_, *φ*_*(1j1)*_), …, (*Y*_*(nj1)*_, *φ*_*(nj1)*_)	(*ΔY*_*(1j1)*_, *φ*_*(1j1)*_), …, (*ΔY*_*(nj1)*_, *φ*_*(nj1)*_)
2	(*Y*_*(1j2)*_, *φ*_*(1j2)*_), …, (*Y*_*(nj2)*_, *φ*_*(nj2)*_)	(*ΔY*_*(1j2)*_, *φ*_*(1j2)*_), …, (*ΔY*_*(nj2)*_, *φ*_*(nj2)*_)

*Y*_*(ijk)*_ denotes the Cq value reported by a qPCR assay for molecular target *j* (*j* = 1, 2, …, or *g*) of sample *i* (*i* = 1, 2, …, or *n*) in group *k* (*k* = 1 or 2). Δ*Y*_*(ijk)*_ denotes normalized Cq for molecular target *j* of sample *i* in group *k*. *φ*_*(ijk)*_ denotes I(*Y*_*(ijk)*_ < C_1_), where C_1_ denotes an assay-specific maximum cycle threshold for quality, clinical, or biological relevance.

As mentioned earlier, there has been a dilemma or disagreement in handling qPCR data with uncertain observations. To address the data analysis challenge, we thoroughly considered scientific and statistical features of qPCR data. The qPCR amplification trajectory has biological parallels, one of which is the growth curve of a non-enveloped virus, adenovirus type 5, in human cells [41]. In the example of adenovirus type 5, after the eclipse and latent periods, the concentration of virus progeny reaches a threshold and virions are released from cells into the extracellular environment. Researchers term the release of virions as viral shedding and often treat it as an event of interest [42, 43]. Likewise, we can treat the reach of the fluorescence threshold in qPCR reactions as an event of interest. Therefore, the data illustrated in Table 1 are like time-to-event data. Considering the qPCR features, we call the observations in Table 1 cycle-to-threshold data to reflect the fact that we are interested in whether the fluorescence threshold is reached and the corresponding amplification cycle if it is reached.

Like in time-to-event studies, we used the conditional rates to reach the threshold to test the null hypothesis of no relationship between the treatment and levels of a target molecule (H_0_). The conditional rate is defined as $\underset{\delta \to 0}{\lim}\frac{1}{\delta }P\left(\Delta \text{y}\leq \Delta \text{Y}\langle \Delta \text{y}+\delta |\Delta \text{Y}\geq \Delta y\right)$ and denoted as λ(ΔY), where ΔY is the normalized cycle outcome. λ(ΔY) sufficiently defines the normalized cycle distribution to reach the fluorescence threshold. If the normalized cycle distribution to reach the fluorescence threshold was the same in the treatment and control groups, our data would support that H_0_: λ_1_(ΔY) = λ_2_(ΔY). The alternative hypothesis can be one-sided, e.g., λ_1_(ΔY) < λ_2_(ΔY), or two-sided, e.g., λ_1_(ΔY) ≠ λ_2_(ΔY). To test the null hypothesis, we need a test statistic. We can translate the test statistic of the exact time-to-event log-rank method to our setting, should the proportional conditional rate, which means λ_1_(ΔY) = *δ* λ_2_(ΔY) where *δ* is a constant, can be assumed. Likewise, we can adapt the generalized version of the exact log-rank method, i.e. exact Fleming-Harrington method, if the proportional conditional rate assumption may be violated [13, 14]. The p-value is obtained with the exact distribution in the exact log-rank or Fleming-Harrington test while it is obtained with a normal distribution with the mean of 0 and standard deviation of 1 in the traditional log-rank test, which is appropriate for studies with a large sample size. We use the exact tests to account for small sample sizes, which are commonly used in preclinical studies and early phases of clinical studies [3, 44–48].

**Fig 2:**
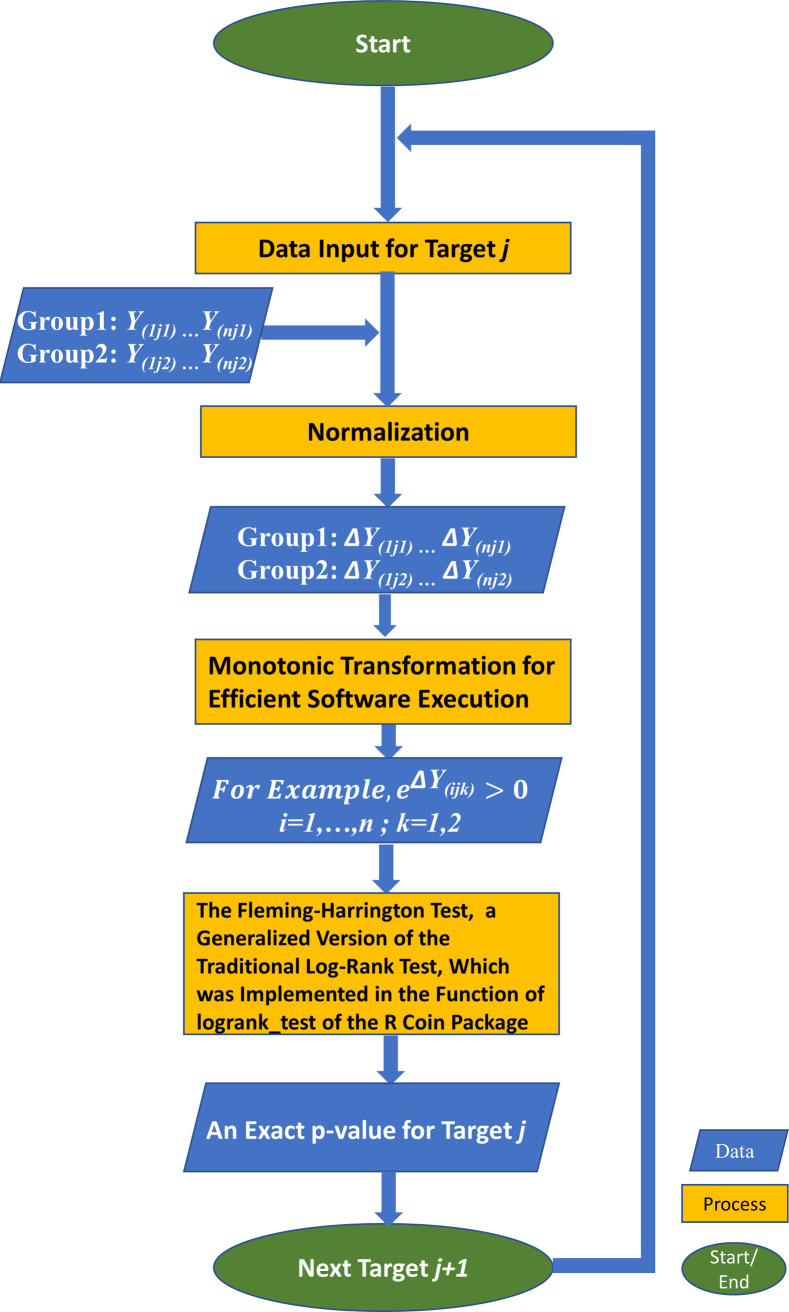
The flowchart to perform CTOT with the R coin package. *Y*_*(ijk)*_ denotes the Cq value reported by a qPCR assay for molecular target *j* (*j* = 1, 2, …, or *g*) of sample *i* (*i* = 1, 2, …, or *n*) in group *k* (*k* = 1 or 2). Δ*Y*_*(ijk)*_ denotes normalized Cq for target *j* of sample *i* in group *k*.

First, we explain the adaption of the exact time-to-event log-rank method to our setting with two comparison groups, e.g., the control and treatment groups, as follows. Suppose that we observe at least one sample reaching the fluorescence threshold at r distinct points in the combined samples of the two groups, i.e., at least one Cq value is observed at each of the r points. With each detection point d (d = 1, 2, 3 …, r), we can organize and tabulate the data as shown in Table 2. For each d, let P_d1_ and P_d2_ be the respective number of molecular targets, e.g., microRNAs, that can be possibly detected at the start of the detection point d in the two groups. Let P_d_ be the sum of P_d1_ and P_d2_. Let O_d1_ and O_d2_ denote the number of molecular targets that are detected at the detection point d in the two groups, respectively. Likewise, let O_d_ be the sum of O_d1_ and O_d2_. Given that O_d_ is detected at d, under the null hypothesis, the variable of O_d1_ given P_d1_, P_d2_, O_d_, and P_d_ follows a hypergeometric distribution, which is $P\left(O_{dk}=o_{dk}|P_{dk},O_{d},P_{d}-O_{d}\right)=\frac{\left(\begin{array}{c} O_{d}\\ o_{dk} \end{array}\right)\left(\begin{array}{c} P_{d}-O_{d}\\ P_{dk}-o_{dk} \end{array}\right)}{\left(\begin{array}{c} P_{d}\\ P_{dk} \end{array}\right)};k=1\;\text{or}\;2$. Therefore, $E\left(O_{dk}|P_{dk},O_{d},P_{d}-O_{d}\right)=\frac{O_{d}}{P_{d}}P_{dk}={\hat{\lambda }}_{d}P_{dk}$, where ${\hat{\lambda }}_{d}$ is an estimate of the overall reaching rate at d. Let *E*_*dk*_ = *E*(*O*_*dk*_|*P*_*dk*_, *O*_*d*_, *P*_*d*_–*O*_*d*_), we can use *μ*_*dk*_ = *O*_*dk*_–*E*_*dk*_ as the kernel of the test statistic. Let *V*_*dk*_ = *var*(*μ*_*dk*_), $U_{D}=\overset{D}{\underset{d=1}{\sum }}\mu_{dk}$ and $V=\sum_{d=1}^{D}V_{dk}$. The p-value of the test statistic $CTOT=\frac{U_{D}}{V^{1/2}}$ can be obtained with the exact distribution via the existing R package, coin [49–51]. In a summary, under the null hypothesis of a common cycle-to-threshold rate for two groups (*k* = 1 or 2), the CTOT test statistic is formed using the sum of the observed minus expected counts over all detected points. As highlighted in the flowchart to perform CTOT with the R coin package (Fig 2), a monotonic transformation that preserves the original order of *ΔY*_*(ijk)*_ for molecular target *j* (*j* = 1, 2, …, or *g*) of sample *i* (*i* = 1, 2, …, or *n*) in group *k* (*k* = 1 or 2), e.g. e^ΔY(ijk)^ > 0, can be applied for efficient execution by a software tool, e.g. the function of logrank_test in the R coin package. It is worth to mention that the exact Fleming-Harrington test in the R coin package calculates optional weights, which are often denoted p and q, for earlier event time and later event time. If both p and q are equal to zero, the exact Fleming-Harrington test is reduced to the exact log-rank test.

**Table 2:** Contingency table on the observations at a detection point.

Group	Potential to be detected	Detected	Undetected (Incomplete)
**1**	P_d1_	O_d1_	P_d1_- O_d1_
**2**	P_d2_	O_d2_	P_d2_- O_d2_
**Total**	P_d_	O_d_	P_d_- O_d_

P_d1_ and P_d2_ denote the number of molecular targets, e.g., microRNAs, that can possibly be detected at the start of the detection point d in Group 1 and Group 2, respectively. P_d_ denotes the sum of P_d1_ and P_d2_. O_d1_ and O_d2_ denotes the number of molecular targets that are detected at the detection point d in Group 1 and Group 2, respectively. O_d_ denotes the sum of O_d1_ and O_d2_.

Table 3 connects CTOT and qPCR data features and common time-to-event parameters. Hazard rate, hazard ratio, median survival time, and the proportion surviving at the end of a study are common time-to-event parameters for interpretation [13, 14, 52, 53]. Correspondingly, the CTOT rate, CTOT ratio, median Cq, and the proportion of uncertain quantifications can also be used to interpret CTOT results. Thus, in addition to p-value, the effect size estimate of the CTOT ratio can be reported in applications of the CTOT method. For researchers who are familiar with time-to-event analysis, the CTOT rate and ratio are good starting points to understand CTOT interpretable parameters. For researchers who are familiar with qPCR data interpretation, the median Cq and proportion of uncertain quantification is a good starting point to understand CTOT’s interpretable parameters.

**Table 3:** Connection between CTOT and qPCR data features and common time-to-event parameters.

Time-to-event parameters	Time-to-event definitions	CTOT parameters	CTOT definitions
Hazard rate	Conditional rate to reach the event (often denoted as λ)	CTOT rate	Conditional rate to reach the fluorescence threshold
Hazard ratio	Ratio of λ_1_ and λ_2_, where λ_j_ is the hazard rate in group j	CTOT ratio	Ratio of λ_1_ and λ_2_, where λ_j_ is the CTOT rate in group j
Median survival time	Time by which 50% of the group of interest have died and another 50% of them have survived	Median Cq	Median of the cycles when targets reach the fluorescence threshold
Proportion surviving at the end of a study	The proportion of the group of interest who have survived at the end of the study	Proportion of uncertain quantifications	Proportion of uncertain quantification by the quality cycle cut-off (C_1_)

### Existing MC, CO, and CNA methods

The MC method sets uncertain Cq values to C_1_ [11, 29, 31, 54–56]. As illustrated in Eq (1), for any *φ*_(*ijk*)_ = 0, i.e. *Y*_(*ijk*)_ ≥ C_1,_ the single value of C_1_ is filled in to obtain the MC normalized Cq value, denoted as *ΔY*_(*ijk*)_*MC*_ in Eq (1), for molecular target *j* (*j* = 1, 2, …, or *g*) of sample *i* (*i* = 1, 2, …, or *n*) in group *k* (*k* = 1 or 2). For other samples with *φ*_(*ijk*)_ = 1, i.e., *Y*_(*ijk*)_ < C_1,_ *Y*_(*ijk*)_ is used to obtain *ΔY*_(*ijk*)_*MC*_. In the literature, MC normalized Cq values are subsequently used to perform analysis with methods designed for continuous outcomes, e.g., ANOVA and t-tests [31, 55]. As mentioned in the Introduction section, without the loss of generalization, we aimed to test the null hypothesis of no relationship between the treatment (or exposure) groups and molecular target levels measured by qPCR. Various types of t-tests have been applied frequently in literature to test the null hypothesis. Statistically, two-independent-groups comparisons were relevant to the setting in this study. Therefore, t-tests for two-independent-groups comparisons were used along with the MC method to illustrate the performance, advantages, and limitations of the MC method.


$$\Delta Y_{\left(ijk\right)\_MC}=\{\begin{array}{c} Y_{\left(ijk\right)}-Y_{\left(irk\right)},\;if\;\varphi_{\left(ijk\right)}=1\\ C_{1}-Y_{\left(irk\right)},\;if\;\varphi_{\left(ijk\right)}=0 \end{array}$$ (1)

In contrast, the CO method excludes the samples with *φ*_(*ijk*)_ = 0 from subsequent analysis [11, 31, 56, 57]. The CO normalized Cq values, for example, *Y*_(*ijk*)_*CO*_ of sample *i* (*i* = 1, 2, …, or *n*) in group *k* (*k* = 1 or 2) in Eq (2), are subsequently used to perform analysis with methods designed for continuous outcomes, e.g. t-tests [31, 56, 57]. In this study, t-tests for two-independent-groups comparisons were used along with the CO method to illustrate the performance, advantages, and limitations of the CO method. The t-test for two-independent-groups comparisons is one of the widely-used tests for qPCR data [2, 58].


$$\Delta Y_{\left(ijk\right)\_CO}=\{\begin{array}{c} Y_{\left(ijk\right)}-Y_{\left(irk\right)},\;if\;\varphi_{\left(ijk\right)}=1\\ \left(excluded\right),\;if\;\varphi_{\left(ijk\right)}=0 \end{array}$$ (2)

The CNA method simply chooses not to analyze the molecular targets with uncertain qPCR data. With the CNA method, should *φ*_(*ijk*)_ = 0 exist for molecular target *j* (*j* = 1, 2, …, or *g*) for any sample *i* (*i* = 1, 2, …, or *n*) in group *k* (*k* = 1 or 2), the molecular target will be excluded from subsequent analysis [20].

### Simulation evaluations and real-world examples

We used simulations to comparatively evaluate the performance of CTOT in analyzing qPCR data that may arise in laboratory experiments [3, 59, 60]. Simulations are important to comparing CTOT’s statistical power with existing analytical methods and verifying the maintenance of type I error rates in CTOT, in which the exact distribution has been utilized to obtain p-values and control type I error rates in the method design stage [49]. Random draws from known distributions are used to efficiently generate data for simulation evaluations. The descriptive statistics, such as range, of the resulting simulated data will be compared with Cq data in literature. In order to generate realistic Cq values in simulations, as illustrated in Table 5, the intercept β_0_ was randomly selected from a normal distribution with the mean of 10 and standard deviation of 2.24, i.e., N(10, 2.24). The effect size of β_1_ was randomly selected from a normal distribution with the mean of 1 and standard deviation of 4.47, i.e., N(1, 4.47). The normalizer was based on a normal distribution with the mean of 25 and standard deviation of 0.45, i.e., N(25, 0.45). The means and standard deviation values were in reference to parametric models fit to a set of rat Cq values and were expected to ensure that the simulated data is reasonably realistic [3]. It is worth to mention that the choices of N(25, 0.45), N(10, 2.24) and N(1, 4.47) are nonexclusive. Researchers can test the methods with any simulation parameters/approaches of choice. Therefore, initial starting values are based on a set of rat Cq values in the data-generation simulation mechanisms, as simulations are often partially based on real data for generalization [49, 59].

The assumption of a normal distribution is usually invoked for well-observed qPCR data in practice, while a t distribution is often reasonably assumed when the sample size is small [2, 56, 57]. Consequently, we simulated microRNAs, including the microRNA normalizer, with Cq values following normal distributions with varying parameters. It is worth noting that, in this setting, the normalized Cq values (ΔCq) are still normally distributed, as the sum or difference of two independent normally distributed random variables is normally distributed and ΔCq is the difference between Cq values of molecular targets and the normalizer in the widely used 2(-Delta Delta C(T)) method [27, 61]. We also used data simulated based on other distributions to evaluate the statistical power of CTOT in detecting differential changes. Different data-generating models were used for the purposes of simulation and method evaluation [59]. Because the distributions of molecular targets in liquid biopsies, such as circulating microRNAs with levels varying, are uncertain or unknown in various biological and environmental conditions, it is necessary to assess how robust CTOT is when the assumption of normality is violated [6, 59]. In addition to normal distributions, we simulated ΔCq data based on extreme value distributions and logistic distributions with varying parameters to assess the reach of CTOT in terms of detecting differential changes with non-normal distributions involved. Compared to normal distributions, the two distribution types may have longer or heavier tails, which may represent the features that are not compatible with normal distributions, e.g., the involvements of rare or completely absent nucleic acids in informative differential changes [1, 13]. As proven by statistical theories, the two distribution types are related to Weibull distributions and log-logistic distributions (S2 Fig). Details regarding the distribution relationships are not included in the scope of this paper. Interested readers may see references on theoretical statistics [61, 62].

In simulations under the log-normal, Weibull, and log-logistic types (Table 4), the comparisons of the performances of the three methods of CTOT, MC, and CO were preceded as: first, the replicate data sets were simulated in the context of a known distribution and population parameters with variation from small treatment effects to strong effects (Table 5). Each data set has two groups of subjects, i.e., the control and treatment (or exposure) groups. The sample size was set to be 5 in each group as it represented the small sample size, which was commonly used in laboratory experiments [44–48, 63, 64].

**Table 4:** One-to-one correspondence of the distributions of simulated ΔCq and e^ΔCq^ data.

Simulation Type	Distribution of e^ΔCq^ Data	Distribution of ΔCq Data
**A**	Log-normal	Normal
**B**	Weibull	Extreme Value
**C**	Log-logistic	Logistic

ΔCq denotes normalized Cq. The natural logarithm of a variable that follows a log-normal distribution is normally distributed. Likewise, the distribution of the natural logarithm of a variable that follows a Weibull distribution is an extreme value distribution. The distribution of the natural logarithm of a variable that follows a log-logistic distribution is a logistic distribution. Therefore, with the natural logarithm transformation, we can obtain normalized Cq values (ΔCq) that follow a normal, extreme value, or logistic distribution by simulation with log-normal, Weibull, or log-logistic distribution, respectively.

**Table 5:** Simulation models with respective density functions and parameters.

y = e^ΔCq^	Log-normal distribution	Weibull distribution	Log-logistic distribution
**Probability Density Function**	$\frac{1}{\text{y}\sigma \sqrt{2\pi }}e^{\left(-\frac{1}{2\sigma^{2}}\left(\log\left(y\right)-\mu_{i}\right)2\right)}\;\text{where}\;\mu i$ = β_0_+*x*_*i*_ β_1_	$\mu_{i}py^{p-1}e^{-\mu_{i}y^{p}}$ where *μ*_*i*_ = e^-*p*(β0+*xi* β1)^	$\frac{\lambda_{i}^{\frac{1}{\gamma }}t^{\frac{1}{\gamma }-1}}{\gamma {\left(1+{\left(\lambda_{i}t\right)}^{\frac{1}{\gamma }}\right)}^{2}}$ where *λ*_*i*_ = e^-(β0+*xi* β1)^
**Parameters For Empirical Power Investigation**	β_0_: 100 random variables from *N*(10, 2.24);		
	β_1_: 100 random variables from *N*(1, 4.47); σ = 1,2		
	*N*(*v*, *η*): Normal distribution with mean *v* and standard deviation *η*		

ΔCq denotes normalized Cq. The natural logarithm of a variable that follows a log-normal distribution is normally distributed. Likewise, the distribution of the natural logarithm of a variable that follows a Weibull distribution is an extreme value distribution. The distribution of the natural logarithm of a variable that follows a log-logistic distribution is a logistic distribution. Therefore, with the natural logarithm transformation, we can obtain normalized Cq values (ΔCq) that follow a normal, extreme value, or logistic distribution by simulation with log-normal, Weibull, or log-logistic distribution, respectively.

Likewise, a common nominal type I error rate of 0.05 was used [3]. Researchers may simulate data with a larger sample size that is compatible with their research context or use a smaller nominal type I error rate. We then applied three analytical methods to analyze each simulated replicate. With the known true relationships in simulations, the type I error rates and statistical power of the three methods were empirically obtained and compared [59].

We simulated 1,000 sample replicates for 300 data scenarios with different distributions or parameter sets [3]. The data from a real-world rat in-vivo microRNA qPCR experiment are used as the base case to obtain the parameter values [3]. Therefore, the 300 scenarios potentially represent 300 microRNAs. We used 300 microRNAs as targets exclusively for illustrative purpose, while limiting the computational burden of simulations. Each of the 1,000 replicates consisted of 10 samples, five of which represented the control group and the other five represented the treated or exposed group. In notation, for each replicate, *Y*_*(ijk)*_ denotes the Cq value reported by a qPCR assay for molecular targets *j* (*j* = 1, 2, …, or 300) of sample *i* (*i* = 1, 2, …, or 5) in group *k* (*k* = 1 or 2). Counting on the 1,000 replicates, this simulated 10,000 samples, each of which had 300 microRNAs as targets. Leveraging an existing simulation R package, survsim, and the relationship between the distributions (Table 4 and S2 Fig), we simulated data using models with varying parameters under 100 log-normal distributions, 100 Weibull distributions, and 100 log-logistic distributions [62, 65]. In total, 300 distributions were used to represent the distributions of 300 microRNAs under various conditions. The simulation models are summarized in Table 5.

The empirical power was calculated based on the following steps: (1) repeatedly simulated Cq data along with group categories under fixed parameter settings (Table 5); (2) analyzed the simulated full data sets using given association tests and compared the resulting p-value of a given significant level, α (e.g., α = 0.05) to determine success (in rejecting the null hypothesis) or failure; and (3) compute the success rate over multiple replicates.

The R statistical software tool was used to perform statistical analysis [66]. Particularly, the existing R packages of survsim and coin were used to perform simulations and apply the CTOT method, respectively [50, 65]. The datasets used and/or analyzed during the current study are available from the corresponding author on reasonable request.

As reported by Silva et al. [3], male and female F344 adult rats were fed a diet containing 0, 30, 60, 120, 180, or 240 ppm each of MEL and CYA for 28 days and terminal blood was collected by cardiac puncture and processed to serum. Total RNA, including microRNAs, was isolated from the rat serum samples using a miRCURY RNA Isolation Kit for Biofluids (Exiqon, Vedbaek, Denmark). TaqMan miRNA assays and an ABI 7900HT Fast Real-Time PCR System (Applied Biosystems by Life Technologies, NY, USA) were used to quantify the microRNAs, including miR-128-3p and miR-210-3p. Five spike-ins (Exiqon) were added at different stages of the experiment for quality control of the RNA extraction and quantitative reverse transcription PCR procedures [3].

## Results

### Simulation results

**Fig 3:**
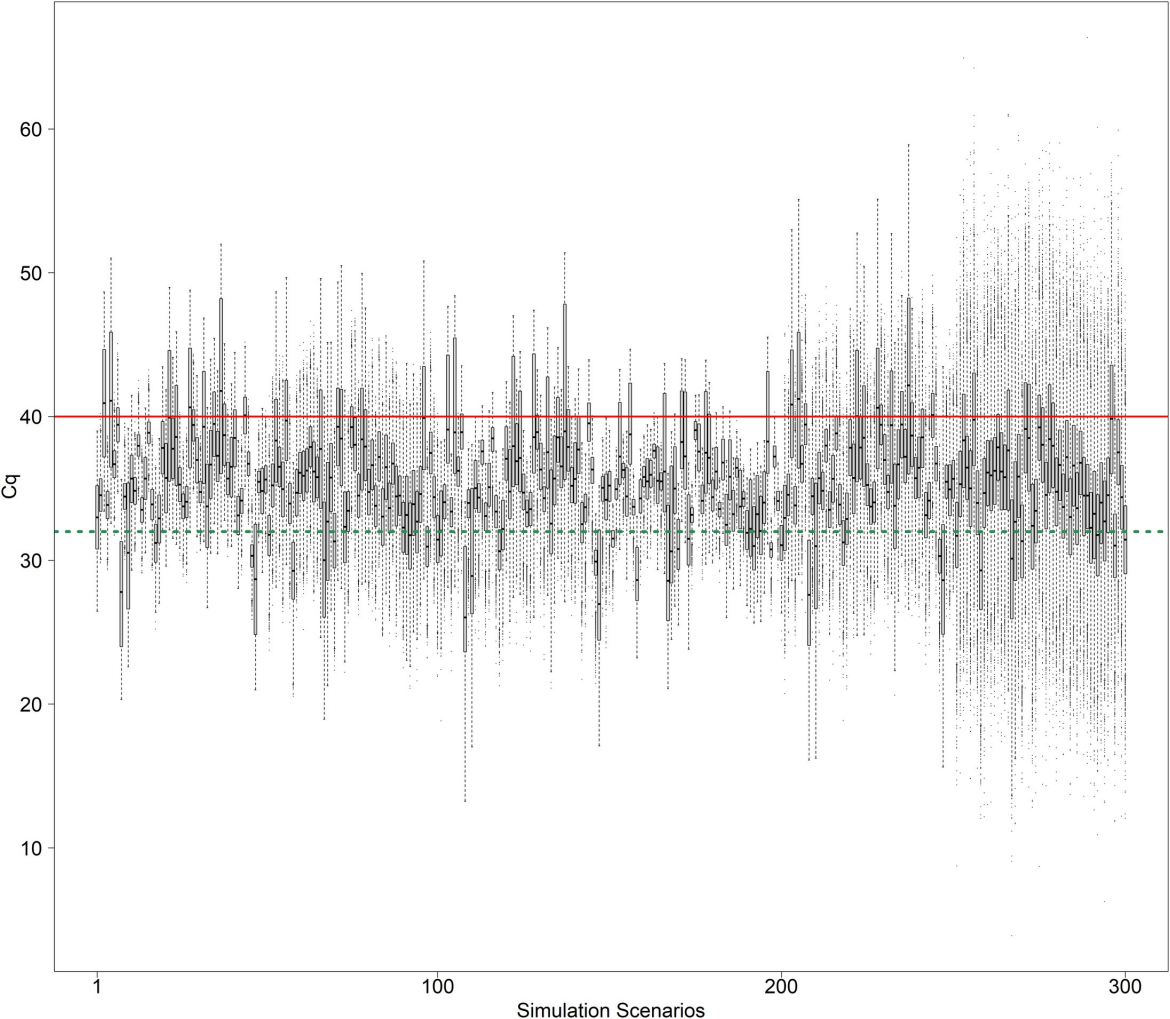
Boxplots of simulated Cq data. The points above the solid line would be uncertainly measured by qPCR should 40 be the cutoff for data quality control or for biological, clinical, or technical concerns in practice. The points above the dash line would be uncertainly measured by qPCR should 32 be the cutoff for quality control or for biological, clinical, or technical concerns in practice.

In this study, without the loss of generalization, we developed and evaluated CTOT, a robust nonparametric cycle-to-threshold method, to test the null hypothesis of no relationship between the treatment (or exposure) groups and molecular target levels measured by qPCR. The alternative hypothesis can be one-sided or two-sided depending on the study purpose [61]. Using simulations, we evaluated the power of CTOT to detect nonzero effects of exposures or treatments with a two-sided alternative hypothesis. Overall, as shown in Fig 3, the simulated Cq data are reasonably in line with realistic biological scenarios (the range of medians: 26.03 to 42.17).

**Fig 4:**
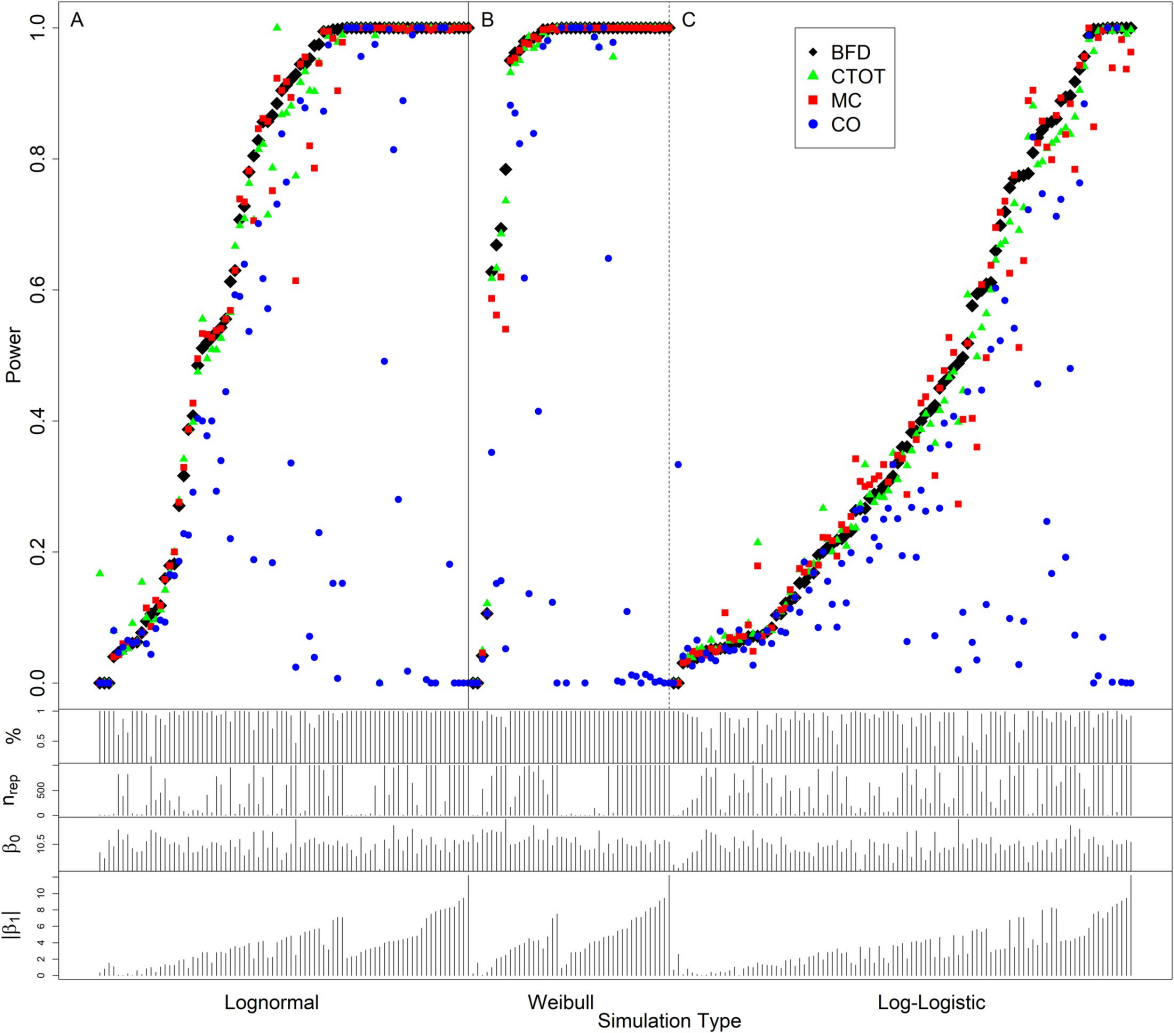
The empirical power of the CTOT, MC, and CO methods compared with that of BFD. BFD stands for the benchmark with full data analyzed with the current standard method, which includes t-tests for two-group comparisons. CTOT stands for the cycle-to-threshold method, while CO denotes the complete-observation method and MC denotes the method that sets uncertain and incomplete observations equal to the assay-specific maximum cycle threshold C_1_. Uncertain qPCR data may occur in one or both groups under comparison. % denotes the percentage of uncertainty that is observed in only one group among the replicates. n_rep_ denotes the number of the replicates with at least one uncertain observation. β_0_ and β_1_ are parameters of the underlying models. |β_1_| is the absolute value of effect size. Panels A, B, and C represent the empirical power of the log-normal, Weibull, and log-logistic simulation type, respectively.

As shown in Fig 3, some simulated Cq data might be inaccurately or incompletely measured by qPCR should 32 or 40 be the cutoff for data quality control or biological/clinical relevance in practice [11, 29, 55, 56]. Without the loss of generalization, we applied the cutoff of 40 in the evaluation via simulation. Fig 4 presents the results of the replicates with at least one uncertain observation, i.e., at least one Cq ≥ 40, in each replicate. The results of log-normal, Weibull, and log-logistic simulations were plotted in the three adjacent panels in Fig 4. In each panel, the empirical power of interest was presented on the vertical axis, while the simulation scenarios were ordered on the horizontal axis. We define the benchmark with full data analyzed using t-tests for two-independent-groups comparisons as BFD. The empirical power of the CTOT, MC, and CO methods as well as BFD was plotted pairwise using different color and symbols. The current standard method to detect a differential change via normalized Cq was the t-test with equal or unequal variances in the two groups of comparison [45, 67, 68]. The simulation scenarios were primarily ordered by the empirical power of BFD in a nondecreasing order. The scenarios were secondarily ordered by the absolute value of the effect size of treatment, i.e., |β_1_| in Table 5. For all simulated scenarios, BFD had the most accurate data values, but some data points could not be fully observed in reality due to LLOQ, LOD, and LOB. Therefore, BFD might not directly pertain to practice. It is necessary to evaluate the effectiveness of analytical methods in compensating for uncertain qPCR data, such as the CTOT, CO, and MC methods, compared with BFD. For simulation scenarios in Fig 4 A, the empirical overall power values of the CTOT and MC methods were slightly lower than that of BFD (empirical overall power: 77.77%, 77.02%, and 79.87%; 95% Monte Carlo CI: 77.35%-78.19%, 76.59%-77.44%, and 79.46%-80.27%, respectively; Table 6). The empirical overall power of the CO method was much lower than that of BFD (empirical overall power: 26.38% and 79.87%; 95% Monte Carlo CI: 25.93%-26.83% and 79.46%-80.27%, respectively; Table 6). Likewise, Fig 4B and 4C indicated that the empirical power of the CTOT and MC methods were better than that of the CO method (Table 6). While the overall power of CTOT seemed similar to that of MC, the overall performance of CTOT was statistically and significantly better than that of MC (improvement in empirical overall power: 0.75% [p-value < 0.001], 1.19% [p-value < 0.001] and 1.56% [p-value < 0.001] for the log-normal, Weibull, and log-logistic simulation types, respectively; Table 6). The improvement was slightly bigger but not diminished when the simulation type changed from log-normal to Weibull or log-logistic with the distribution of Cq not normally distributed. This indicates that CTOT is a robust method without restrictive distributional assumptions. This observation is consistent with the nonparametric nature of CTOT. In contrast, the CNA method would not analyze the simulated comparisons with observations that could be incompletely measured in practice [20, 31].

**Table 6:** Empirical overall power of the CTOT, MC, and CO methods with analysis on benchmark data. BFD stands for the benchmark with full data analyzed with the current standard method, which includes t-tests for two-group comparisons. CTOT stands for the cycle-to-threshold method, while CO denotes the complete-observation method and MC denotes the method that sets uncertain observations equal to the assay-specific maximum cycle threshold C_1_.

Simulation Type	Evaluation	BFD	CTOT	CO	MC
**Log-normal (n = 37,100)**	Correct Decisions	29,630	28,853	9,788	28,573
	Empirical Overall Power (95% Monte Carlo CI)	79.87% (79.46%-80.27%	77.77% (77.35%-78.19%)	26.38% (25.93%-26.83%)	77.02% (76.59%-77.44%
**Weibull (n = 25,861)**	Correct Decisions	24,043	23,879	5,912	23,572
	Empirical Overall Power (95% Monte Carlo CI)	92.97% (92.66%-93.28%)	92.34% (92.01%-92.66%)	22.86% (22.35%-23.37%)	91.15% (90.80%-91.50%)
**Log-logistic (n = 54,293)**	Correct Decisions	27,496	26,339	9,475	25,491
	Empirical Overall Power (95% Monte Carlo CI)	50.64% (50.22%-51.06%)	48.51% (48.09%-48.93%)	17.45% (17.13%-17.77%)	46.95% (46.53%-47.37%)

**Fig 5:**
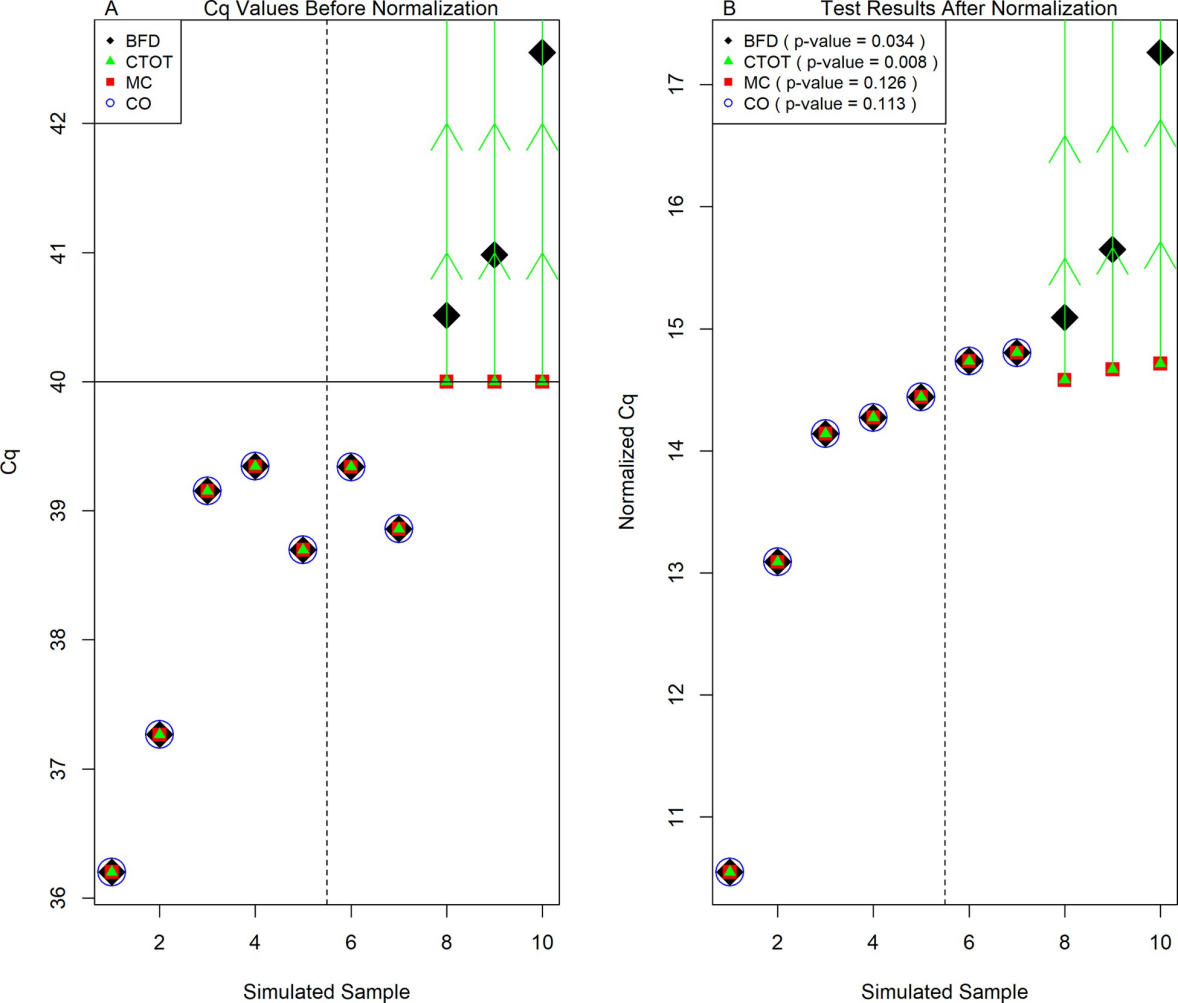
An Example to Illustrate the Issue of Potential False Negatives of MC and CO. (A) The original Cq data simulated with a normal distribution (corresponding to the log-normal simulation type in Table 2, *β*_0_ = 13.35 and *β*_1_ = 2.06; the corresponding empirical power of BFD is 0.80). (B) The normalized Cq data with the BFD, CTOT, MC, or CO methods applied. The filled diamonds denote the Cq data with BFD. BFD stands for the benchmark with full data analyzed with the current standard method, which include t-tests for two-group comparisons. The filled triangles denote the Cq data with CTOT, the cycle-to-threshold method. The vertical green arrows indicate the ranges uncertain observations belong to, e.g., being greater than or equal to the assay-specific maximum cycle threshold C_1_. The filled squares denote the Cq data with MC, the method that sets uncertain and incomplete observations equal to C_1_. The maximum quality cycle threshold C_1_ = 40 is highlighted with a horizontal solid line. The open circles denote the Cq data with CO, the complete-observation method. The first five simulated samples belong to Group 1. The second five simulated samples belong to Group 2. The vertical dash line separates Groups 1 and 2.

Fig 5A illustrates the application of the MC, CO, CTOT, and BFD methods on a simulated Cq data set. Fig 5B illustrates the limitations of the MC and CO methods compared to CTOT and BFD. As indicated in a study by Zhuang et al. [69], the CO method reduces the sample size, while the MC method can strikingly decrease both Cq and normalized Cq, both of which tend to bring false negatives. The utilization of range values in CTOT can mitigate the deficit of MC as the ranges permit inclusion of the true values of Cq. As shown in Fig 5B, with uncertain Cq values set to 40, one of the common maximum quality qPCR cycle cutoffs [22, 29], MC failed to detect the differential change that was detected by BFD (p-values: 0.126 and 0.034, respectively). Considering the range of Cq ≥ 40, CTOT could correctly detect the difference (p-value: 0.008; Fig 5B), should the significance level of 0.05 be applied, as described in the Materials and Methods section of this article. In addition, should the CO method be applied, 3 out of 5 observations in the second group would be removed from analysis and the difference between the two groups would be considered undetectable (p-value: 0.113; Fig 5B), while it is detectable by CTOT (p-value: 0.008) when the realistic Cq cutoff, 40, is applied [22, 29]. Therefore, CTOT may mitigate the deficits of CO and MC in data analysis.

**Fig 6:**
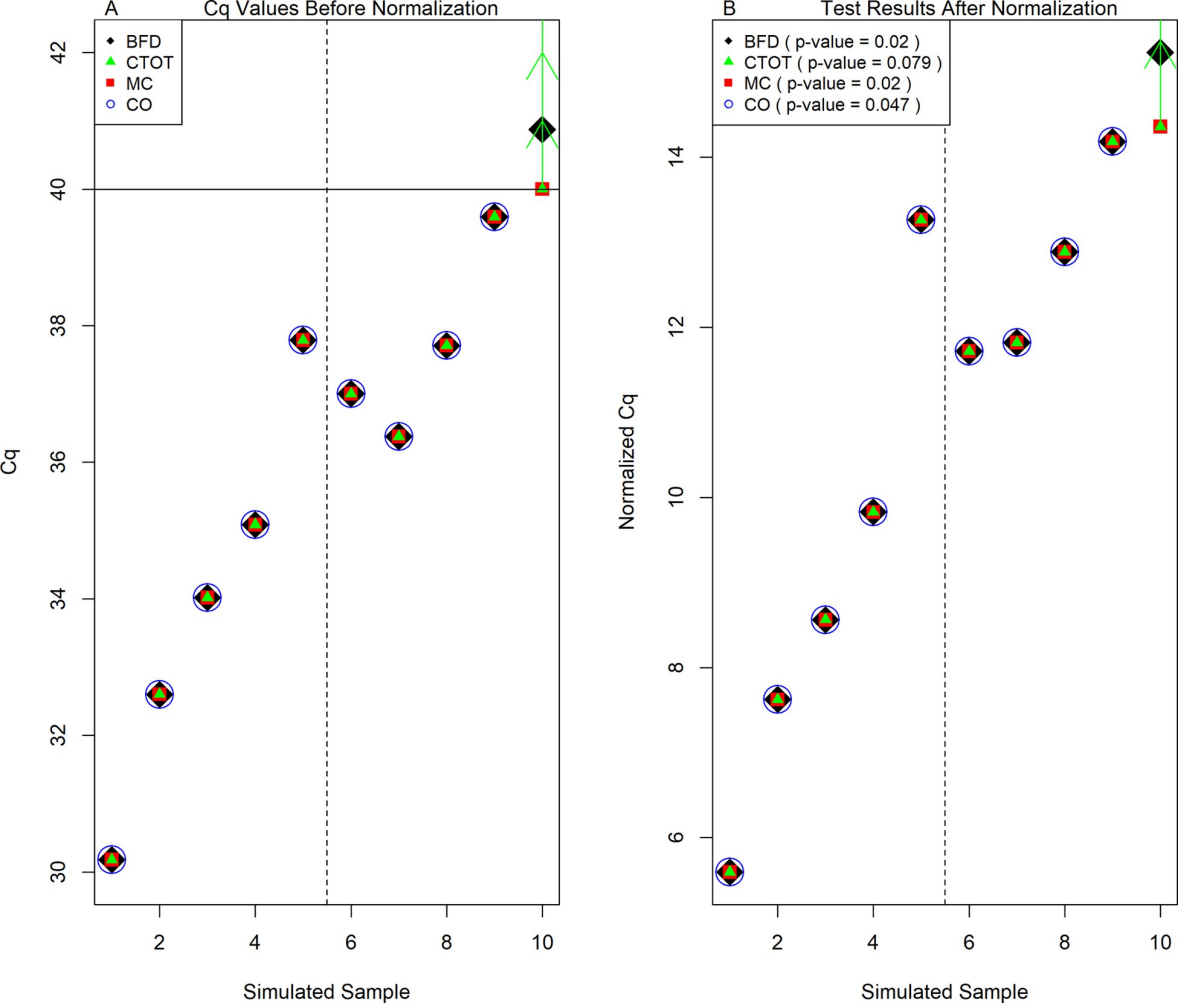
An example to illustrate differences of the MC, CO, and CTOT methods. (A) The original Cq data simulated with a normal distribution (corresponding to the log-normal simulation type in Table 5, *β*_0_ = 8.47 and *β*_1_ = 4.65; the corresponding empirical power of BFD is 0.91). (B) The normalized Cq data with the BFD, CTOT, MC, or CO methods applied. The filled diamonds denote the Cq data with BFD. BFD stands for the benchmark with full data analyzed with the current standard method, which include t-tests for two-group comparisons. The filled triangles denote the Cq data with CTOT, the cycle-to-threshold method. The vertical green arrows indicate the ranges uncertain observations belong to, e.g., being greater than or equal to the assay-specific maximum cycle threshold C_1_. The filled squares denote the Cq data with MC, the method that sets uncertain and incomplete observations equal to the assay-specific maximum cycle threshold C_1_. The maximum quality cycle threshold C_1_ = 40 is highlighted with a horizontal solid line. The open circle denoted CO, the complete-observation method. The first five simulated samples belong to Group 1. The second five simulated samples belong to Group 2. The vertical dash line separates Groups 1 and 2.

In addition, it is interesting to note that a low frequency of uncertain observations in qPCR data may not deteriorate the performance of CO and MC (compared with BFD), especially when the sample Cq values arise from an underlying normal distribution and uncertain observations are not influential points that cause foremost changes in the analysis and decisions [70]. For example, there is merely one Cq observation higher than 40 in the scenario illustrated in Fig 6. Both CO and MC methods established statistical significance in this scenario (p-value: 0.047 and 0.020, respectively). Should the maximum quality qPCR cycle be 40, CO would exclude the single uncertain observation from analysis. MC would set the Cq value larger than 40 to be 40 and obtain the p-value of 0.02, similar that of BFD. However, the p-value of CTOT was 0.079. A larger p-value occurs in this case because CTOT is a nonparametric method, while CO and MC are parametric methods. Like many other nonparametric methods, the test statistic of CTOT is constructed in terms of ranks among the normalized Cq values, as described in the Materials and Methods section. The t-tests used in CO and MC focus on comparing the mean values of the two groups with the normality assumption after excluding or imposing a value to the uncertain Cq observation. Realistically, the sample mean values of the original and normalized Cq observations tend to be unknown in the samples with uncertain observations. When there are many qPCR observations censored at C_1_, the lowest Cq value that can be quantified with acceptable certainty for the context of use, the sample mean values of the original and normalized Cq observations tend to be biased downward by the MC and CO methods [71]. Instead of a number, the range an uncertain observation belongs to, e.g., being larger than or equal to 40, is often available as illustrated in Fig 1, the amplification qPCR plot. With information in the ranges of uncertain observations, CTOT focuses on the order or ranks of the observations to construct the test statistics and compare the groups. Thus, CTOT can be more robust and powerful in many situations (Table 6 and Fig 5). However, CTOT might be less powerful than the parametric methods, CO, and MC, in a case when there is minor uncertainty and the underlying Cq distribution is a normal distribution, like the situation illustrated in Fig 6.

**Fig 7:**
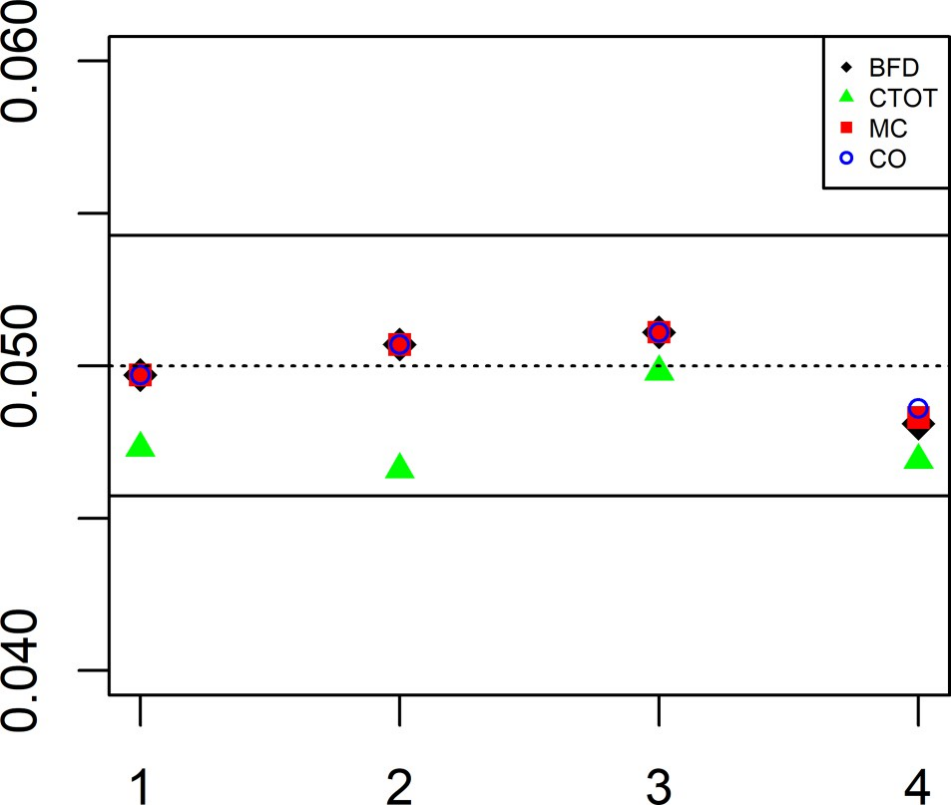
Empirical type I error rates of CTOT, BFD, CO, and MC methods. CTOT stands for the cycle-to-threshold method. BFD stands for the benchmark with full data analyzed with the current standard method, which includes t-tests for two-group comparisons. CO denotes the complete-observation method and MC denotes the method that sets uncertain observations equal to the assay-specific maximum cycle threshold C_1._ In the simulation, C_1_ is set to be 40.ΔCq followed normal distributions and e^ΔCq^ followed log-normal distributions. Parameter Set 1: β_0_ = 5, σ = 1; Parameter Set 2: β_0_ = 10, σ = 1; +Parameter Set 3: β_0_ = 5, σ = 2; and Parameter Set 4: β_0_ = 10, σ = 2 with the parameterization listed for log-normal distribution in Table 2.

We obtained the results of 10,000 replicates for each log-normal simulation with β_1_ equal to 0, β_0_ equal to 5 or 10, and *σ* equal to 1 or 2, respectively (Fig 7). The empirical type I error rates of CTOT, MC, and CO as well as the analysis on benchmark data (BFD) were within the Monte Carlo 95% confidence interval of 0.046 to 0.054 corresponding to a nominal type I error rate of 0.05. Similar to what we observed in simulated data for power analysis (Fig 3), some of the simulated Cq data for type I error investigation might not be accurately measured by qPCR should 40 be the cutoff for data quality control or biological/clinical relevance in practice. Thus, some replicates involved uncertain data in simulation. We then determined the type I error rate of CTOT with the replicates that had at least one uncertain observation. With the 710 replicates that satisfied the criterion of at least one uncertain observation, the type I error rate of CTOT was 0.042. The type I error rate of CTOT is satisfactory as it is within the Monte Carlo 95% confidence interval from 0.034 to 0.066, which corresponds to the 710 replicates with uncertain observations and the nominal type I error rate of 0.05 [72].

### Real-world application results

**Fig 8:**
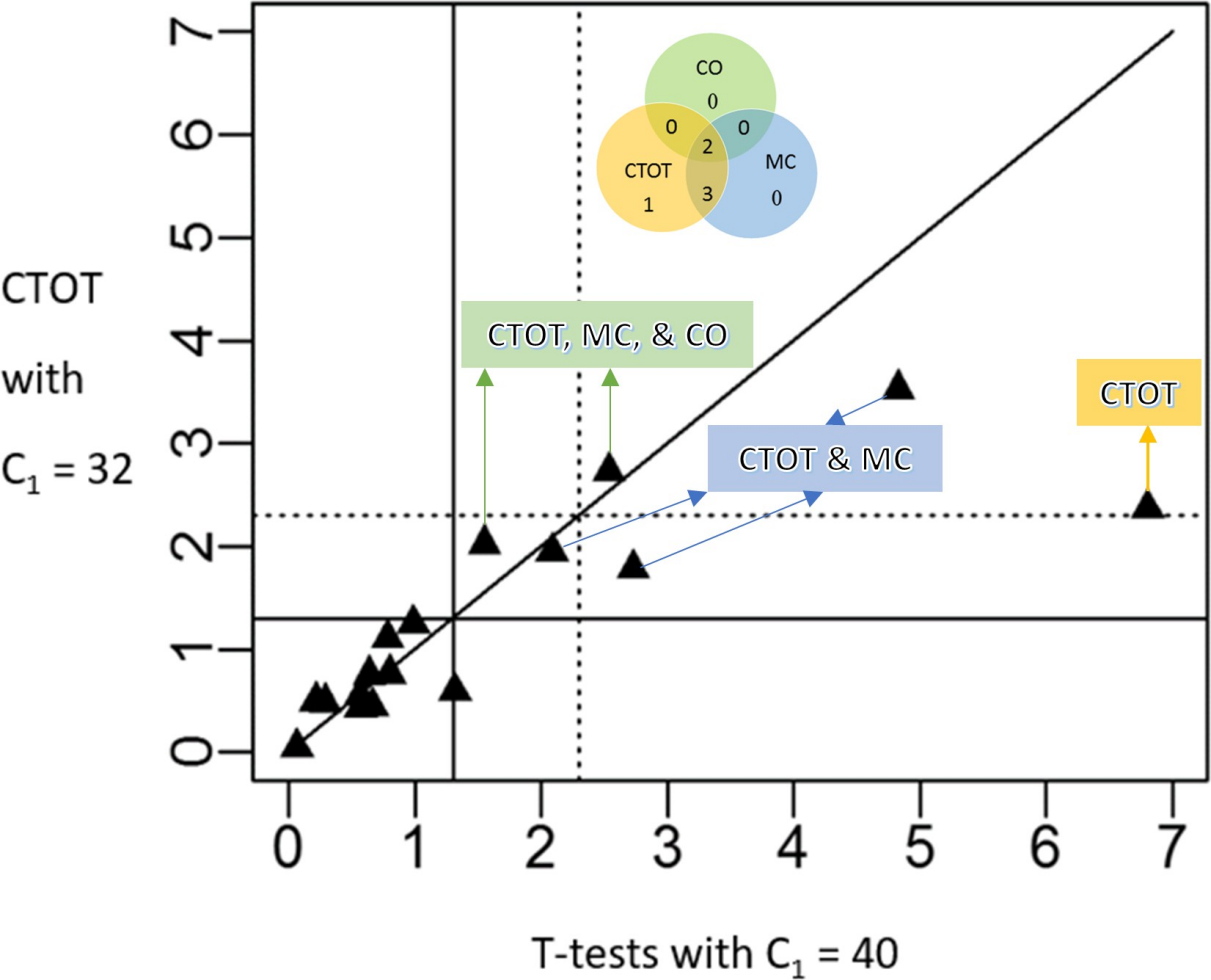
Comparison of the statistical significance between t-tests with C_1_ = 40 and CTOT with C_1_ = 32. The sensitivity analysis was performed on 17 two-group comparisons on rat serum microRNAs miR-210-3p and miR-128-3p, where there was at least one uncertain observation in either of the two groups of comparison [3]. The p-values based on t-tests and CTOT (cycle-to-threshold method) are plotted in a -log_10_ scale on x-axis and y-axis, respectively. C_1_ denotes an assay-specific maximum cycle threshold for clinical, quality, or biological relevance, e.g., the cycle number that corresponds to LLOQ (lower limit of quantification). The solid lines are set at p-value = 0.05 and the dashed lines are set at p-value = 0.005. The inset Venn diagram illustrates statistically significant differences of levels of circulating microRNAs between control and treated groups, applying the CTOT, MC, or CO method and a maximum quality cycle threshold of C_1_ = 32 to the data reported by Silva et al. [3].

For the application illustration, we applied CTOT to a published experimental data set. The application involved sensitivity analysis with the two Cq cutoff values most commonly used in the literature, 32 and 40 [3, 22, 23, 55, 56, 73]. In general, sensitivity analysis assesses how sensitive a model or analysis result is to its data or input variables, on which the model or analysis result is built [74, 75]. Silva et al. [3] reported that serum microRNAs, including miR-128-3p and miR-210-3p, were affected in a dose-dependent manner by nephrotoxic doses of melamine (MEL) and cyanuric acid (CYA) in male and female rats. All Cq values of microRNAs miR-128-3p and miR-210-3p were less than 40, the default Cq cutoff value for quality control in the qPCR software tool (Applied Biosystems Sequence Detection Systems (SDS) software, version 2.4.1). Others have suggested using the Cq cutoff of 32, i.e., C_1_ = 32, for circulating microRNAs [55, 73]. With a Cq cutoff of 32 applied to the serum miR-128-3p and miR-210-3p qPCR data, three of the 20 two-group comparisons between a control group and one of the exposure groups of 30, 60, 120, 180, and 240 ppm MEL and CYA on male and female rats had certainly determined data, i.e., Cq < 32 in both the control and exposure groups, while the remaining 17 had at least one uncertain observation in either of the two groups of comparison. As simulation results showed that CTOT could detect differential changes in many situations without excess type I errors, we applied CTOT to the data on the 17 two-group comparisons. As shown in Fig 8, the p-values of six out of 17 tests were found to be less than 0.05 by both CTOT with C_1_ = 32 and t-tests with the original data (all original Cq values < 40). The one found to be marginally significant by the t-test with the original data (p-value = 0.048) was not confirmed to be statistically significant by CTOT with C_1_ = 32, should the significance level of 0.05 be applied. The p-values of all other 10 out of 17 tests were found to be larger than 0.05 by both CTOT with C_1_ = 32 and t-tests with the original data (all original Cq values < 40). Thus, CTOT was able to confirm all similarities between the control and exposure groups and detected all but one significant difference, regardless of a more conservative Cq cutoff value, with the qPCR data on serum microRNAs miR-210-3p and miR-128-3p [3].

As discussed, CTOT might help ascertain some differential expressions that would be missed otherwise by CNA, CO, MC, or all of these statistical methods. In the application illustrated in the scatter plot of Fig 8, CTOT confirmed six of the seven differential changes (p-values ≤ 0.05) that were detected by t-tests with C_1_ = 40 [3]. In contrast, MC confirmed five out of seven differential changes, while CO confirmed only two out of seven differential changes (Fig 8). Therefore, consistent with our simulations (Table 6 and Figs 4 and 5), CTOT can alleviate both the disadvantages and consequences, especially mitigatable false negatives, of CO, MC, and CNA by leveraging the range information of uncertain observations. Table 7, in the Conclusions section, summarizes the advantages and disadvantages of CTOT, CNA, CO, BFD and MC.

**Table 7:** Methods to analyze qPCR data with uncertain observations.

Method	Outcome Data Type in Normalization and Analysis	Advantages	Disadvantages
CTOT	Two outcome variables: A continuous variable, i.e., observed Cq values or contextual Cq cut-off C_1_, and a binary variable, i.e., being observed or censored at C_1_	It is a robust nonparametric method with good Type I error and power properties. CTOT overcomes the decrease of sample size caused by CO and the artificially decreased variance of Cq in MC. The utilization of exact distribution in inferences helps researchers obtain the minimum sample size and uses resources in a cost-effective way, which are often required in preclinical studies and early phases of clinical studies	When the underlying data distribution is known, its statistical power may be less strong than the parametric methods that match the underlying data distribution, as CTOT is a nonparametric method
CNA	Not applicable since no analysis is performed	False positive rates are zero	True positive rates are zero. No evaluations on candidate biomarkers with uncertain Cq values
CO	One outcome variable: A continuous variable, i.e., completely observed Cq values only with the incomplete observations deleted from analysis	It is useful when the reduced sample size is still large enough to discover or verify a differential change and the true Cq data distribution is a normal distribution	It reduces the sample size of a study, which may decrease statistical power in the evaluation of a candidate biomarker. In addition, its statistical power is limited when the true Cq data distribution is not a normal distribution
BFD	One outcome variable: A continuous variable, i.e., simulated/assumed Cq values that are observed or unobserved in reality due to contextual Cq cut-off C_1_	It may be used as a benchmark in simulations	BFD does not directly pertain to practice because some data points cannot be fully observed in reality due to LLOQ (a lower limit of quantification), LOD (limit of detection), and LOB (limit of blank)
MC	One outcome variable: A continuous variable, i.e., completely observed Cq values and contextual Cq cut-off C_1_, to which the incomplete observations were set	The filled-in values with contextual Cq cut-off C_1_ may still allow the discovery or verification of a differential change, especially when the true Cq data distribution is a normal distribution	MC may bring false discoveries when the Cq data distribution is skewed by the simple imputation method or when the true Cq data distribution is not a normal distribution

## Discussion

Several analytical technologies, such as qPCR, enable the measurement of molecular targets for biomarker discoveries, early diagnosis and treatment, as well as better patient and disease management. However, some data may be uncertain but still contain useful information for differential changes between controls and treatments of interest. Because of LOB, LOD and LLOQ, some researchers focus on differential changes that involve well-observed data in both groups. While this strategy has advanced biological discoveries, it may bring research irreproducibility and limit knowledge from further accumulating and evolving in differential detection with emergent qPCR-based technologies, e.g., qPCR-based liquid biopsies.

In some studies, researchers have sufficient historical data and have accumulated knowledge on the data distribution type of the target population, e.g., the normal distribution. Researchers may choose the most appropriate statistical methods accordingly. However, it is possible that the underlying data distribution is not well-known and, therefore, lacks corresponding parametric methods. In some studies, we do not know or are unsure of the underlying data distributions. If there are many absent levels in samples, the distribution of Cq values would not be a normal or t distribution as the probability of going to infinite in a normal or t distribution is extremely low. When the underlying model assumptions are not valid, the results of the parametric models tend to be invalid and less robust than those of nonparametric models that do not assume an underlying distribution of the data. In these studies, a robust nonparametric or a semi-parametric method will be more appropriate as it does not use or heavily assume the underlying distribution of the target population.

A nonparametric method may fit the current context with uncertain qPCR data better because the concentration distributions of molecular targets under exploration, such as microRNAs in liquid biopsy, are unclear under various biological and environmental conditions. In the investigation via simulation, the empirical power of our CTOT method is very close to that of the benchmark with full data analyzed with the current standard method, t-tests. The results of CTOT are consistent with the theoretical expectations for a nonparametric method and performs better than the parametric methods when the underlying distribution is very different from the normal or t distribution. Therefore, should CTOT detect a significant differential change, the result will be robust as the method does not assume an underlying distribution.

Furthermore, CTOT pushes the analysis limit and helps overcome the limitations of commonly used analytic strategies, e.g., the CNA, MC, and CO methods. CTOT fully accounts for outcomes, binary, continuous, or ranges, and aligns with the principle of normalization in analysis. The utilization of exact distribution in inferences helps researchers obtain the minimum sample size and uses resources in a cost-effective way [49]. In preclinical studies and early phases of clinical studies, the sample sizes are often required to be as minimal as possible. With CTOT, researchers can use the valid and robust analytical method to analyze incomplete qPCR quantification and do not have to choose not to analyze (CNA) such incomplete data. CTOT overcomes the decrease of sample size caused by CO and the artificially decreased variance of Cq in MC. It can also overcome the restrictiveness in distributional assumptions of the current standard method, e.g. two-group t-tests [61]. Thus, it leverages qPCR technology and contributes to the reproducibility of both certain and uncertain qPCR data.

If we model part of the outcomes, i.e., the binary outcome on the reach of the fluorescence threshold, and apply an analytical method, such as logistic regression, we will retain information on the occurrence of uncertainty or incompleteness due to low or absent levels of molecular targets, but we lose information on the timing of events. Recently proposed methods, such as the expectation-maximization-based imputation and the Markov chain Monte Carlo-based hierarchical model methods, impute missing or censored observations with observed data and statistical assumptions [11, 12]. The imputation methods are expected to perform well in the situations when the statistical assumptions are not violated and information in observed data is sufficient to impute missing or censored observations well enough for study objectives [11, 12, 76]. There remain the situations when the true Cq values of the incomplete observations cannot be imputed well from the observed data (e.g., actually, Cq = infinity), or when it is insensible to assume the statistical assumptions, e.g., normality or other parametric assumptions [2, 31]. A nonparametric method may fit better than a parametric method that uses or assumes the underlying distribution of the population, as the concentration distributions of molecular targets under exploration, such as microRNAs, are unclear under various biological and environmental conditions. Simulation results indicate that CTOT, a nonparametric method that incorporates ranges in analysis, may robustly detect differential expression when the statistical assumptions, e.g., normality or other parametric assumptions, are violated.

The utilization of p-values for decision making has been discussed recently in both the literature and the research community. Research reproducibility is vital to the regulatory sciences in the biopharmaceutical area. In this study, we used the power of 80% and the nominal type I error rate of 0.05 in simulations. In sensitivity analysis, the alpha level of 0.05 was used as it is compatible with existing real-world data [3]. The type I error rate of 0.05 and sample size of 5 in a group are commonly used in rat in vivo studies to explore exposure effects on microRNAs [3, 44–48]. The type I error rate and sample size are usually decided to achieve a sensible power level, e.g., 80%, to detect a biologically meaningful population effect of a treatment on a microRNA with reasonable resources. Literature has shown that, with a low power, such as 0.52, p-values become unreliable for inference as the type I error rate may be inflated [77]. Meanwhile, the p-value cutoff for decision making should not be reduced too small to detect true differences with a sensible statistical power, if the sample size is fixed; otherwise, the power will be decreased with false negatives increased. A low alpha level without a compatible sample size may decrease power unfavorably and increase false negatives (S3 Fig). For example, should we change the alpha level from 0.05 to 0.005, the decreased power may be as low as 0.1, given that the other factors are fixed. Should the alpha level in the sensitivity analysis be set to be 0.005, only three tests have a lesser p-value (Fig 8). Researchers may use a lower alpha level, e.g. 0.005, and/or, a higher power, e.g. 90% with a compatible sample size and study purpose [39]. Therefore, like many studies, the use of the p-value, as well as the choice of the p-value cutoff for the study to detect interesting differential changes in molecular targets with the qPCR technology, depends on several interrelated factors, including the study question, target population (e.g. the effect size and standard deviation in the target population), available resources (e.g. sample size), and acceptable decision thresholds (e.g. type I error rate and power) [78].

The CTOT method presented here is designed for detecting differential changes with relative quantification of qPCR data, which directly compares groups using normalized Cq data [3, 20, 79]. Absolute quantification can be obtained once the raw qPCR data are compared with pre-determined standard curves [60, 80]. More reagents are needed for standard dilutions to create a standard curve and standard dilutions may bring errors [79]. Furthermore, when the differential changes between groups are of interest, absolute quantification and the determination of the exact levels of DNA input are not necessarily needed, as relative quantification can determine the differential changes with reference molecular targets and samples [28, 60, 79]. Even in well-conducted absolute quantification studies, uncertain data may still be observed as left-truncated, e.g. <25 international units per mL (IU/mL) for plasma HCV RNA [1]. The CTOT method cannot be directly used to analyze the qPCR absolute quantification for differential changes. However, statistical methods that deal with left-truncated or interval-censored data may be adapted or leveraged [12, 81, 82].

One limitation of this study is that it assumes errorless pre-PCR preparation and reliable real-time qPCR reactions. An example of a pre-PCR preparation limitation is hemolysis of blood samples, which may bring unwanted effects when assessing transcript levels in serum and plasma samples [26, 83]. CTOT is currently not designed to account for errors in pre-PCR preparation and qPCR reactions. Prior knowledge and experimental approaches may be used to check whether it is sensible to assume errorless pre-PCR preparation and reliable real-time qPCR reactions. For example, to take into account the extent of blood hemolysis, researchers may classify samples as being hemolyzed if their absorbance at 414 nm exceeded a value of 0.2 [26]. A thorough discussion on pre-PCR preparation and real-time qPCR reactions are out of the scope of the present study. Interested readers may refer to published guidelines and literature [7, 20, 26, 84].

Our study addresses a data analysis challenge when using qPCR data that can be encountered, for example, in the face of a new biomarker detection method with microRNAs in liquid biopsies. The data analysis challenge comes with auxiliary information that all missing or censored data are larger than or equal to the assay-specific maximum qPCR cycle threshold, which is denoted C_1_ in the paper, in the scenarios with errorless pre-PCR preparation and reliable real-time qPCR reactions. An auxiliary variable contains information about missing data but is not needed if there are no missing data. CTOT leverage auxiliary information on C_1._ In addition, the application of CTOT is illustrated with analysis of microRNAs, but the method itself is not microRNA-specific and may be fit for other molecular targets, e.g. viral DNA and mRNAs, as long as uncertain qPCR data are observed for group comparisons and qPCR technology is reliably utilized with good practice [85].

## Conclusions

Biomarker detection and validation with liquid biopsies have faced an analytical challenge due to the overall low levels of nucleic acids in biofluids. In this study, we focus on the identification or validation of cell-free biomarkers in biofluids with qPCR technology, particularly those that represent good practice with errorless pre-PCR preparation and reliable, real-time qPCR reactions. Existing commonly used approaches, including CO, CNA, and MC, exclude samples with uncertainly determined qPCR Cq data, exclude groups with uncertainly determined qPCR Cq data, or set the uncertainly determined qPCR Cq values as all equal to an assay-specific Cq cutoff, e.g., C_1_ in this study. These approaches are inadequate to consider all available information or have made a strong but questionable assumption on samples. In reference to how qPCR Cq data are technically obtained, the challenge to validate the population-level distributions of the Cq values with uncertain observations, and unique Cq data features, e.g., uncertainly determined observations that have true Cq values greater than or equal to C_1_, we designed a novel nonparametric statistical method, CTOT, to improve biomarker detection and validation for liquid biopsies. Our simulations and applied real-world examples show that CTOT represents a new approach and framework for future studies to detect and validate biomarkers with informative but uncertain qPCR Cq values. Table 7 summarizes the advantages and disadvantages of CTOT, CO, CNA, and MC as well as BFD, which was used in simulations. Compared with the three commonly used methods of CO, CNA, and MC, the greatest advantage of CTOT is that it is more robust and powerful to detect remarkable differential changes that involve at least one group that tend to have very low levels of nucleic acids, thus resulting in incomplete or uncertain qPCR data. In general, the more authentic differential changes are identified regarding biological processes of interest, the higher the chances of discovery of safe and effective therapeutics and diagnostic devices (or tests).

## Supporting information

S1 Fig Literature on the topic of qPCR and microRNAs in web of science. The search result covers the period from 1/1/2016 to 12/31/2020. The literature search was performed in the Web of Science Core Collection, which covered over 1.7 billion references and over 30,000 indexed journals [21]. Specifically, we formed one search with keywords and the Boolean operators of OR and AND. The keyword of microRNA and the alternative spelling of miRNA were combined in the search using the Boolean operator of OR. The Boolean operator of AND was further used to restrict the search to include literature on qPCR and microRNA. We obtained search results with “TS = (microRNA OR miRNA) AND TS = (qPCR OR qRT-PCR)” and with the Advanced Search capability in the Web of Science, where TS denoted topic and was a search field tag.
(TIF)

S2 Fig Theoretical distribution relationships used in simulation. Three proofed distribution relationships are used in simulation. Solid lines with arrows represent transformations from one distribution to another. NLT stands for a natural logarithm transformation. ET stands for exponential transformation. For example, the natural logarithm of a variable that follows a log-normal distribution is normally distributed. Likewise, the distribution of the natural logarithm of a variable that follows a Weibull distribution is an extreme value distribution. The distribution of the natural logarithm of a variable that follows a log-logistic distribution is a logistic distribution. Both NLT and ET are monotonic transformations that preserve the order of the original data.
(TIF)

S3 Fig Power analysis of two-sided t-test for two independent groups. The sample size is 5 in each group. The population mean *μ*1 varies from 20 to 30 in Group 1, while the population mean *μ*2 varies from 32 to 40 in Group 2. For simplicity, the population standard deviation is fixed to be 1 in each group. The significance levels (alpha) are set to be 0.001, 0.005, 0.05, or 0.2 for analytical illustration.
(TIF)

S1 Table A tabular summary of power analysis of two-sided t-test for two independent groups. The sample size is 5 in each group. The population mean mu1 varies from 20 to 30 in Group 1, while the population mean mu2 varies from 32 to 40 in Group 2. For simplicity, the population standard deviation is fixed to be 1 in each group. The significance levels (alpha) are set to be 0.001, 0.005, 0.05, or 0.2 for analytical illustration.
(CSV)

S2 Table The empirical power of the CTOT, BFD, CO, and MC methods by parameter sets. BFD stands for the benchmark with full data analyzed with the current standard method, which includes t-tests for two-group comparisons. CTOT stands for the cycle-to-threshold method, while CO denotes the complete-observation method and MC denotes the method that sets uncertain and incomplete observations equal to the assay-specific maximum cycle threshold C_1_. Uncertain qPCR data may occur in one or both groups under comparison.
(CSV)

S1 Text R codes for Figs 5 and 6. (TXT)

## Abbreviation list

BFD: benchmark full data
C_1_: assay-specific maximum qPCR cycle threshold
CI: statistical confidence interval
CNA: choose not to analyze method
cDNA: complementary deoxyribonucleic acid
CO: complete-observation method
Cq: qPCR quantification cycle
CTOT: cycle-to-threshold method
CYA: cyanuric acid
LLOQ: lower limit of quantification
LOB: limit of blank
LOD: limit of detection
MC: maximum quality qPCR cycle threshold method
MEL: melamine
MIQE: minimum information for publication of quantitative real-time PCR experiments
mRNA: messenger ribonucleic acid
qPCR: quantitative real-time polymerase chain reaction
RT: reverse transcription
