# Supplementary figures and images for: A robust biostatistical method leverages informative but uncertainly determined qPCR data for biomarker detection, early diagnosis, and treatment
Source: PLoS One. 2022 Jan 31;17(1):e0263070. doi: 10.1371/journal.pone.0263070 (PMC8803186; doi:10.1371/journal.pone.0263070)

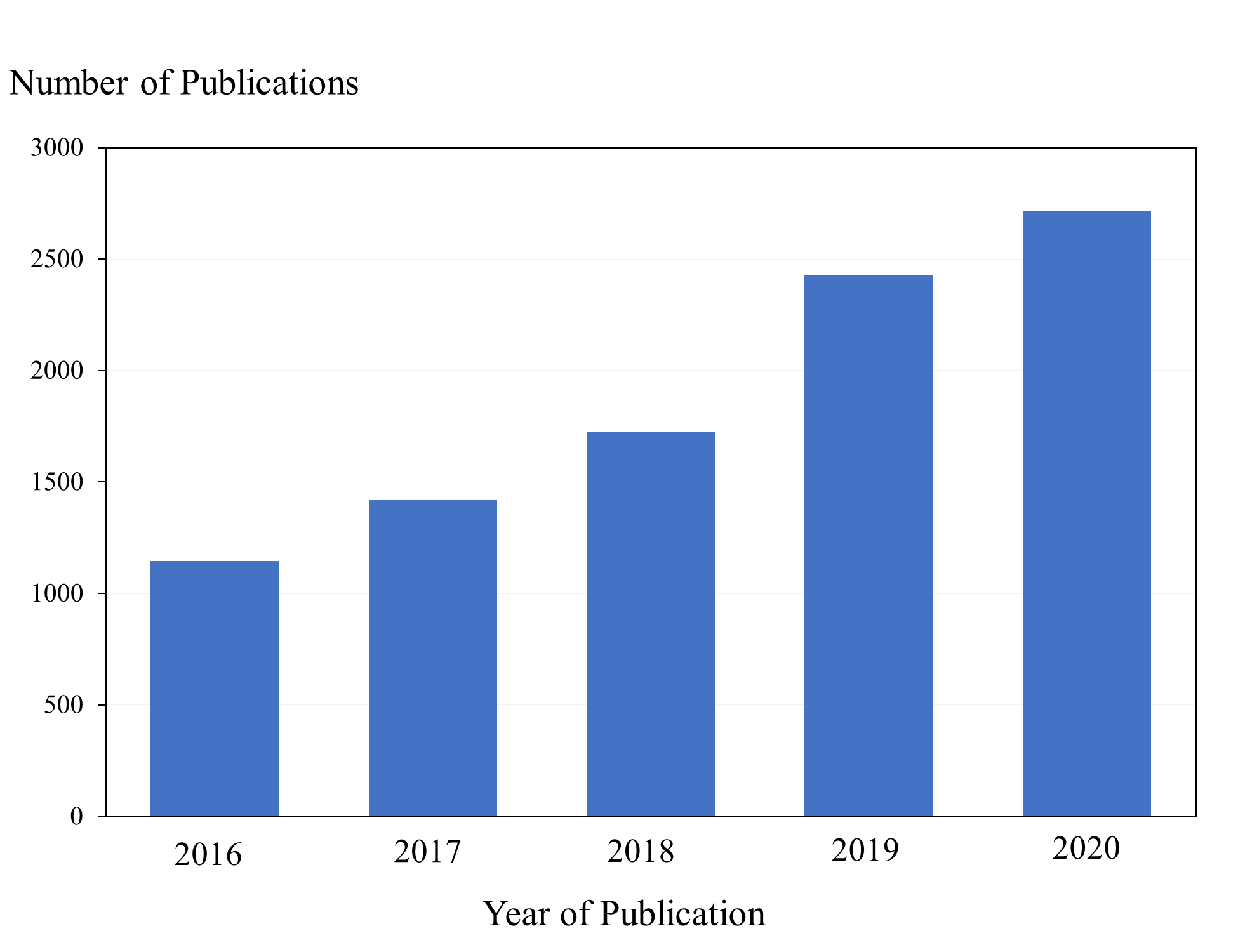

Supplement: S1 Fig — The search result covers the period from 1/1/2016 to 12/31/2020. The literature search was performed in the Web of Science Core Collection, which covered over 1.7 billion references and over 30,000 indexed journals [21]. Specifically, we formed one search with keywords and the Boolean operators of OR and AND. The keyword of microRNA and the alternative spelling of miRNA were combined in the search using the Boolean operator of OR. The Boolean operator of AND was further used to restrict the search to include literature on qPCR and microRNA. We obtained search results with “TS = (microRNA OR miRNA) AND TS = (qPCR OR qRT-PCR)” and with the Advanced Search capability in the Web of Science, where TS denoted topic and was a search field tag. (TIF) [file pone.0263070.s001.tif]

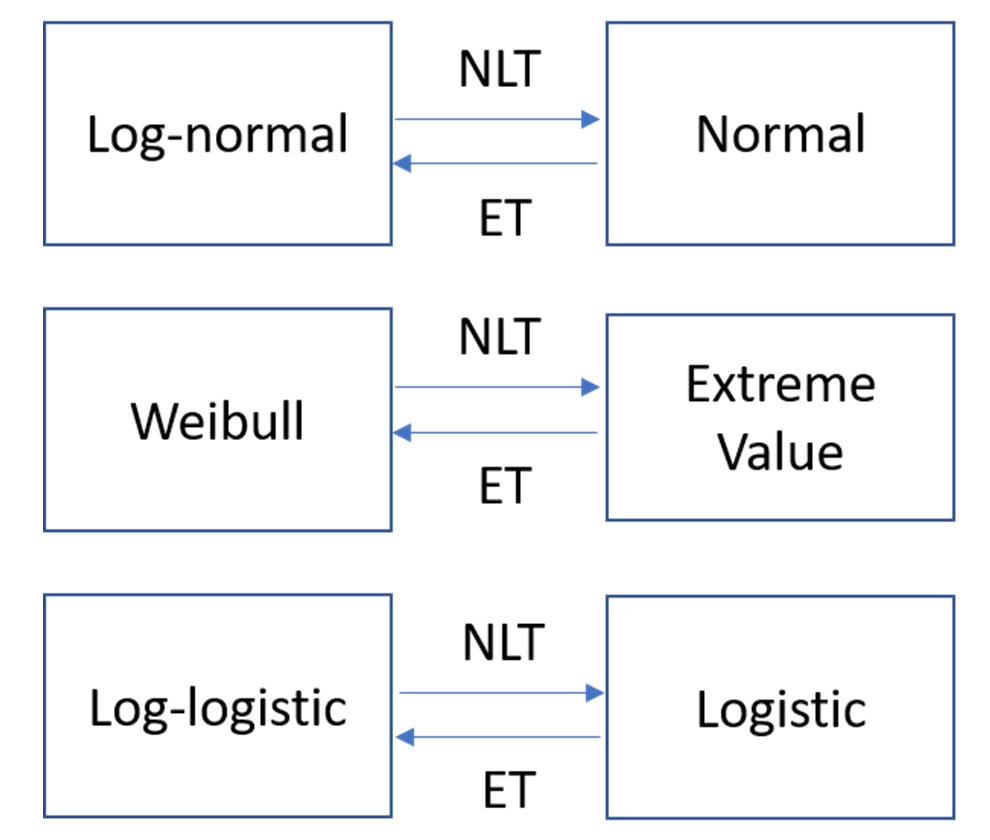

Supplement: S2 Fig — Three proofed distribution relationships are used in simulation. Solid lines with arrows represent transformations from one distribution to another. NLT stands for a natural logarithm transformation. ET stands for exponential transformation. For example, the natural logarithm of a variable that follows a log-normal distribution is normally distributed. Likewise, the distribution of the natural logarithm of a variable that follows a Weibull distribution is an extreme value distribution. The distribution of the natural logarithm of a variable that follows a log-logistic distribution is a logistic distribution. Both NLT and ET are monotonic transformations that preserve the order of the original data. (TIF) [file pone.0263070.s002.tif]

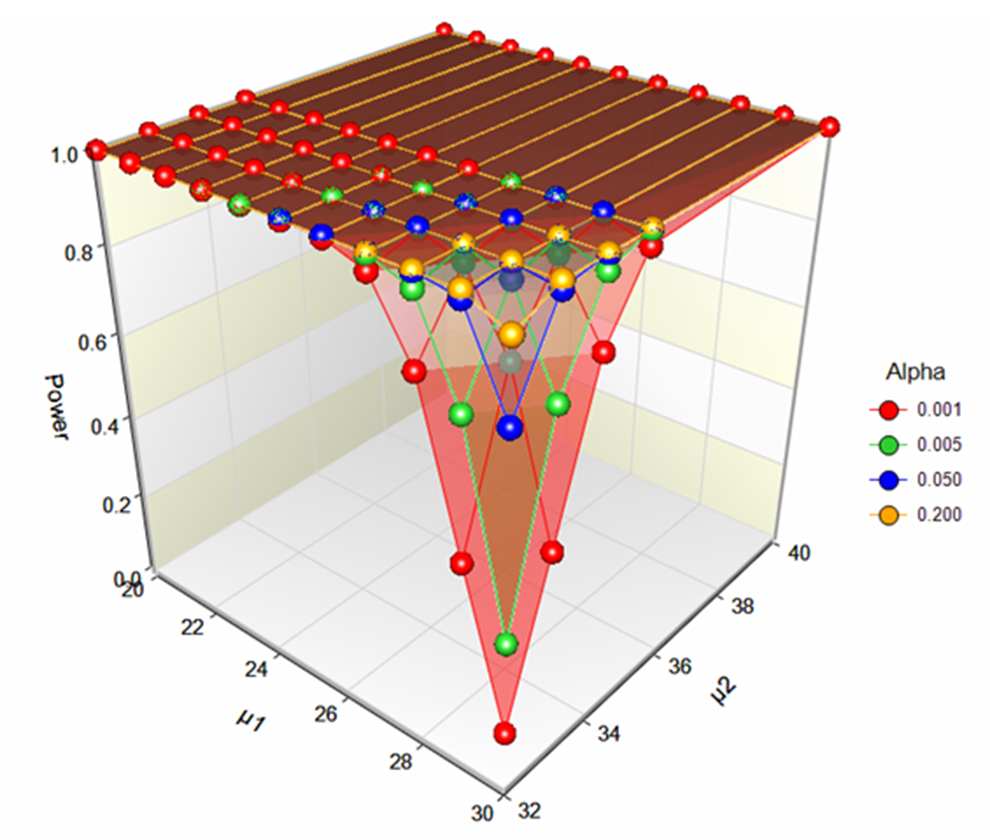

Supplement: S3 Fig — The sample size is 5 in each group. The population mean μ1 varies from 20 to 30 in Group 1, while the population mean μ2 varies from 32 to 40 in Group 2. For simplicity, the population standard deviation is fixed to be 1 in each group. The significance levels (alpha) are set to be 0.001, 0.005, 0.05, or 0.2 for analytical illustration. (TIF) [file pone.0263070.s003.tif]
